# The rice *pds1* locus genetically interacts with partner to cause panicle exsertion defects and ectopic tillers in spikelets

**DOI:** 10.1186/s12870-019-1805-z

**Published:** 2019-05-15

**Authors:** Qigui Jiang, Yindi Zeng, Baiyang Yu, Weijian Cen, Siyuan Lu, Peilong Jia, Xuan Wang, Baoxiang Qin, Zhongquan Cai, Jijing Luo

**Affiliations:** 1 College of Life Science and technology (State Key Laboratory for Conservation and Utilization of Subtropical Agro-bioresources), Guangxi University, Nanning, 530004 China; 2 Institute of New Rural Development, Guangxi University, Nanning, 530004 China; 3 Agriculture College, Guangxi University, Nanning, 530004 China

**Keywords:** Rice, Sheathed panicle, Aberrant spikelets, High-order tillering, Panicle tillering, Fine mapping, Genetic interaction

## Abstract

**Background:**

Rice (*Oryza sativa* L.) is a staple food crop worldwide. Its yield and quality are affected by its tillering pattern and spikelet development. Although many genes involved in the vegetative and reproductive development of rice have been characterized in previous studies, the genetic mechanisms that control axillary tillering, spikelet development, and panicle exsertion remain incompletely understood.

**Results:**

Here, we characterized a novel rice recombinant inbred line (RIL), *panicle exsertion defect and aberrant spikelet* (*pds*). It was derived from a cross between two *indica* varieties, S142 and 430. Intriguingly, no abnormal phenotypes were observed in the parents of *pds*. This RIL exhibited sheathed panicles at heading stage. Still, a small number of tillers in *pds* plants were fully exserted from the flag leaves. Elongated sterile lemmas and rudimentary glumes (occurred occasionally) were observed in the spikelets of the exserted panicles and were transformed into palea/lemma-like structures. Furthermore, more interestingly, tillers occasionally grew from the axils of the elongated rudimentary glumes. Via genetic linkage analysis, we found that the abnormal phenotype of *pds* manifesting as genetic incompatibility or hybrid weakness was caused by genetic interaction between a recessive locus, *pds1*, which was derived from S142 and mapped to chromosome 8, and a locus *pds2*, which not yet mapped from 430. We fine-mapped *pds1* to an approximately 55-kb interval delimited by the markers **pds-4** and **8 M3.51**. Six RGAP-annotated ORFs were included in this genomic region. qPCR analysis revealed that Loc_Os080595 might be the target of *pds1* locus, and *G1* gene might be involved in the genetic mechanism underlying the *pds* phenotype.

**Conclusions:**

In this study, histological and genetic analyses revealed that the pyramided *pds* loci resulted in genetic incompatibility or hybrid weakness in rice might be caused by a genetic interaction between *pds* loci derived from different rice varieties. Further isolation of *pds1* and its interactor *pds2*, would provide new insight into the molecular regulation of grass inflorescence development and exsertion, and the evolution history of the extant rice.

**Electronic supplementary material:**

The online version of this article (10.1186/s12870-019-1805-z) contains supplementary material, which is available to authorized users.

## Background

In higher plants, mature body architecture is established by the iterative formation of lateral organs on the main axes of plants via genetic control of the spatiotemporal initiation of axillary meristems (AMs). During postembryonic growth, in the vegetative stage, AMs are initiated in the leaf axils, from which the axillary buds are formed. At the transition from the vegetative to the reproductive phase of plant growth, the shoot apical meristem (SAM) acquires inflorescence meristem (IM) identity and initiates lateral branch or flower development. In rice, the AMs initiated in the leaf axils develop into tillers on the main culms, whereas the axillary primordium produced by the IM gives rise to ten or more primary branches. Then, the IM degenerates, leaving a degenerated point at the base of the uppermost lateral branch of the rice inflorescence, or panicle (Additional file 1: Figure S1A, D) [1]. During the development of young rice panicles, branch primordia are formed in the axils of bracts (small degenerate leaves). Higher-order AMs are sequentially initiated from the primary branch meristems. The few meristems at the base of the primary branch acquire secondary branch meristem identity and develop into secondary panicle branches, while the other AMs grow into lateral spikelets. Finally, the apical meristems of the primary and secondary branches are transformed into terminal spikelets (Additional file 1: Figure S1B, C, D) [1]. During rice spikelet development, the spikelet meristem (SPM) differentiates a series of modified leaves, including a pair of rudimentary glumes, sterile lemmas, fertile glumes (lemma and palea), and floral organs, including lodicules, stamens, carpel, and stigmas, which are subtended by the pair of fertile glumes and called the floret [2]. The floret number per spikelet varies among grass species. In the extant cultivated rice varieties, only a single floret is subtended by the lemma and palea of the spikelet. Following the maturation of reproductive organs (stage In8), the uppermost internode of the tiller rapidly elongate and the inflorescence protrudes from the sheath of flag leaf [3].

In the development of spikelets, the mutants *frizzy panicle* (*fzp*), *leafy hull sterile1* (*lhs1*), *supernumerary bract* (*SNB*), *degenerative palea* (*dep*)/*abnormal floral organs* (*afo*), *long sterile lemma* (*g1*), and *lateral floret 1* (*lf1*) exhibits panicle architecture and spikelet morphology alterations in rice [2, 4–10]. The genes underlying the mutant phenotypes have been isolated and shown to be involved in the maintenance and/or control of the SPM identity. The mutation of these genes results in the ectopic homeotic transformation of the AMs in the spikelet. On the one hand, some mutated genes ectopically transform the floral organs in the spikelets. For example, a palea/lemma-like (P/LL) structure is ectopically formed in the presumptive position of the rudimentary glumes and/or sterile lemmas. This phenotype can be seen in the spikelets of the *lhs1*, *snb*, and *g1* mutants [4, 6, 8]. On the other hand, the mutants *fzp*, *dep*/*fao*, and *lf1* ectopically produce non-spikelet organs in the spikelets [2, 5, 9, 10]. Mutation of the *FZP* gene leads to the formation of AMs in the axils of the rudimentary glumes instead of a transition from the SPM to the floret meristem. These ectopically transformed AMs either arrest or develop into higher-order branches. *FZP*, the rice ortholog of the maize *BD1* gene, encodes an ERF transcription factor [2, 5]. A dominant mutation of *LF1* unexpectedly produces lateral florets in the axils of the sterile lemmas, causing an alternate phyllotaxy in the *lf1* mutant that results in a ‘three-floret’-like spikelet [10]. More intriguingly, the *pho* phenotypes are caused by double mutation of both *OsMADS15* and *OsMADS1* genes, resulting in the transformation of rice spikelets/flowers into juvenile plantlets and subsequently switching the reproductive pattern from sexual to asexual [9]. This mutation phenotype termed pseudovivipary. *OsMADS15* mainly functions in inhibiting the formation of SAM in incipient floral primordium, while *OsMADS1* mainly involves in promoting the determinacy of floral meristem (FM). Although these two genes play different roles in rice panicle development, they work cooperatively to determine FM, and the double mutations of them in *pho* mutant result in a stable inflorescence reversion [9]. The pseudovivipary phenomenon also can be seen in the overexpression of *OsLEC1* gene in rice [11].

Regarding to the phenotypes of internode elongation, numerous mutants have been well characterized in rice. In general, two classes of mutants were identified for internode elongation. One class is internode-enhanced mutants that increase the plant height of rice. For example, the mutation of *EUI1* gene, resulting in the elevation of bioactive level of GAs in rice plant, the mutant exhibited an extremely elongated uppermost internode and significantly increased its plant height [12, 13]. In contrast, short internode mutants are another class, which leads to a dwarf phenotype. Among these, some mutants showed no uppermost internode elongation at heading stage, on which the panicles were fully or partially enclosed in the leaf sheath. Recent report about two shortened uppermost internode mutants *sui1–1* and *sui1–2* showed that *sui1–1* exhibited a short uppermost internode and a partly sheathed panicle, whereas, *sui1–2* showed an extremely shortened uppermost internode and a fully sheathed panicle. *SUI1* gene is located on chromosome 1 and encodes a putative phosphatidyl serine synthase (PSS) family protein. Furthermore, the shorted internode phenotype of *sui1* is GA insensitive [14, 15].

Thus far, genetic and molecular analyses have identified several genes that control the development of grass inflorescences [2, 5, 7, 10, 16–19]. Interestingly, some of these genes not only play pivotal roles in inflorescence development but also influence the course of development in organs other than the inflorescence [5, 16–18, 20–23]. In short, these genes have pleiotropic effects on plant development. For example, the *teosinte branched1* (*tb1*) gene, underlying a QTL involved in maize domestication, affects inflorescence sex, ear size, branch length and tiller number [16]. In rice, the *APO1* mutation causes numerous defects throughout the life cycle of rice, such as a short plastochron, abnormal phyllotaxis of primary branches, transformation of stamens to lodicules, etc., in addition to resulting in a precocious transition from the IM to SPM [17, 18]. *ASP1* gene also has pleiotropic effects on rice development. Mutation in *ASP1* causes alterations in branching pattern and phyllotaxy, aberrant spikelet morphology, and the release of axillary bud dormancy, thereby resulting in the ectopic development of axillary shoots from the upper nodes of the main culms [23]. The regulatory mechanisms underlying the pleiotropic effects of these genes on plant development have been of great interest to biologists.

Heterosis has been widely applicating in hybrid breeding of crop species such as rice, barley, and rapeseed, and tremendous contribution has been made to enhance agricultural yield because hybrid plants possess superior traits, for example, better adaptability, greater uniformity, and improved abiotic and biotic stress tolerance, etc. [24]. However, heterosis is not always observed in the hybrid plants, whereas hybrid weakness was also observed in some cases of cross combination. In general, hybrid weakness is manifested as the retarded growth, the disorder and disturbance of the hybrid development pattern [25]. Several study cases about hybrid weakness have been reported in crops, either at the interspecific or intraspecific [26–29]. For instance, the pyramiding of *Hwi1*, a locus which is specific to wild rice (*Oryza rufipogon*), and *Hwi2*, a locus that is predominantly distributed in *indica* rice, results in the activation of autoimmune response in the basal nodes of hybrids, and therefore interrupting root formation and then impairing shoot growth. These indicated that genetic incompatibility results in hybrid weakness [29].

In this study, we characterized a RIL, *panicle exsertion defect and aberrant spikelet* (*pds*), which possesses the features of hybrid weakness because many developmental events were disturbed in rice life cycle. The most conspicuous aspect of the *pds* phenotype is its extremely shortened uppermost internode, resulting in a fully or partially sheathed and aborted panicle. Moreover, in the spikelet of a few exserted panicles, the rudimentary and/or sterile glumes are ectopically transformed into P/LL structures, and pseudovivipary (panicle tillering) was observed in the axils of spikelets as well. To characterize the gene(s) underlying the abnormal phenotypes, in present study, we fine-mapped major locus, *pds1*, on chromosome 8 by positional cloning. This locus, genetically interacted with its interactor, has a major effect on the developmental alterations in the *pds* line.

## Results

### The identification of RIL *pds* with abnormal phenotypes

RIL *pds* was observed in an F_2:3_ family derived from a cross between two *indica* rice cultivars, 430 and S142 with normal plant architecture and normal panicle development. To exclude the possibility of *pds* phenotype that was generated by a natural mutation, 20 more different F_2_ lines with normal panicle development and exsertion were planted to produce F_2:3_ families (36 plants per population). Of which, the phenotypic segregation was observed in 6 lines. The result indicated that *pds* phenotype does not cause by natural mutation. Further, the phenotypes of *pds* were stably observed for 6 consecutive generations indicating that the loci underlying *pds* line is homozygous and stably controls its abnormal phenotype.

### Elongation defect in the uppermost internode of the *pds*

**Fig. 1:**
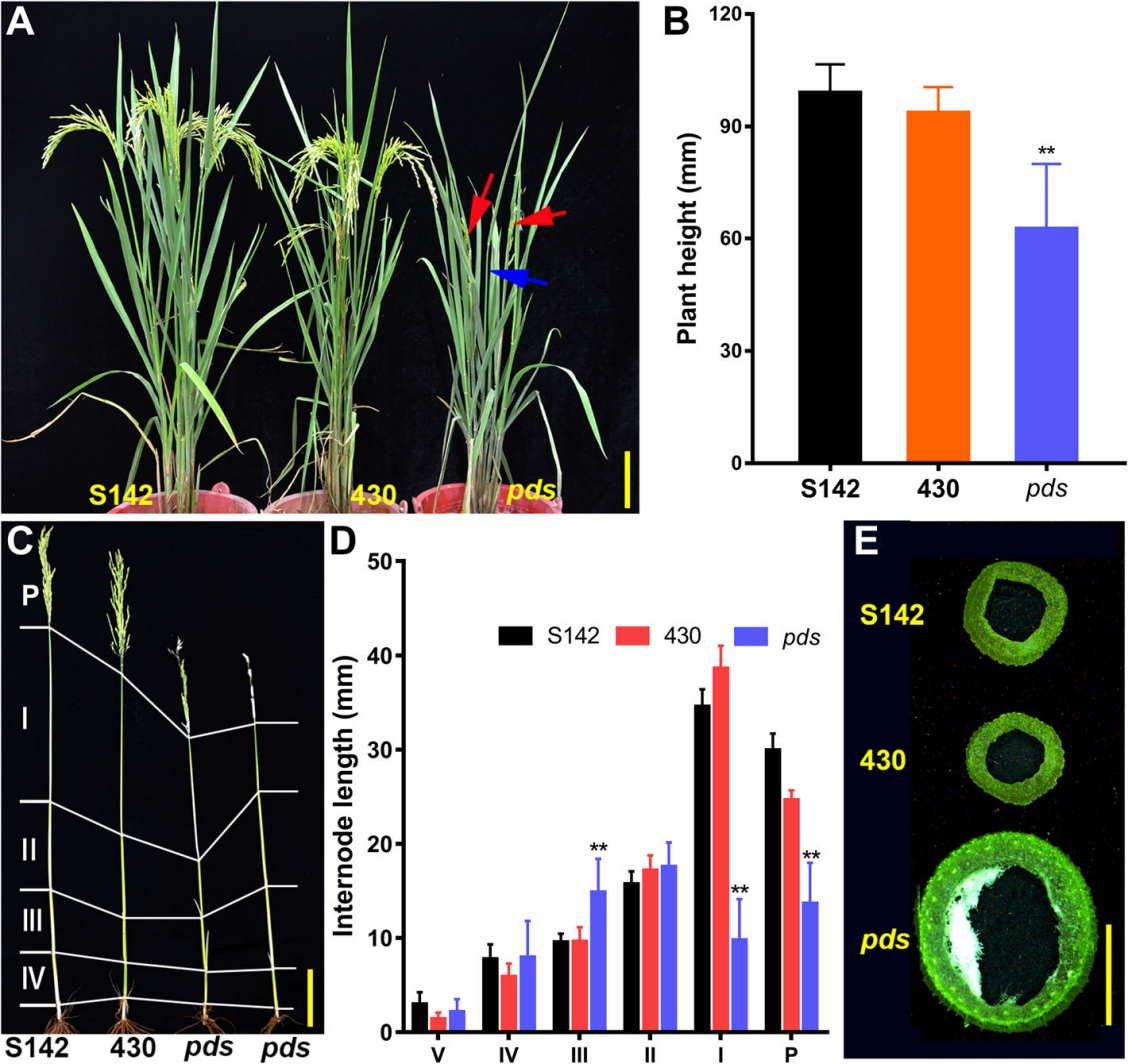
Phenotypic comparison of *pds* and its parental plants (S142 and 430). **a** *pds* plants (right) and parental plants (left and middle) at grain-filling stage. **b** Plant heights of S142, 430, and *pds* plants. **c**, **d** Comparisons of the internode lengths of S142, 430, and *pds* plants. **e** Comparison of the thickness of the uppermost internodes of S142, 430, and *pds* plants. The red arrows in (**a**) indicate the partial exsertion of the *pds* panicles; the blue arrow indicates the exsertion defect of the *pds* panicles. Error bars in (**b** and **d**) indicate the mean ± sd. ^∗∗^Significant difference at *P* < 0.01 compared with the controls according to Student’s *t*-test. Bars = 10 cm in (**a** and **c**) and 5 mm in (**e**)

**Fig. 2:**
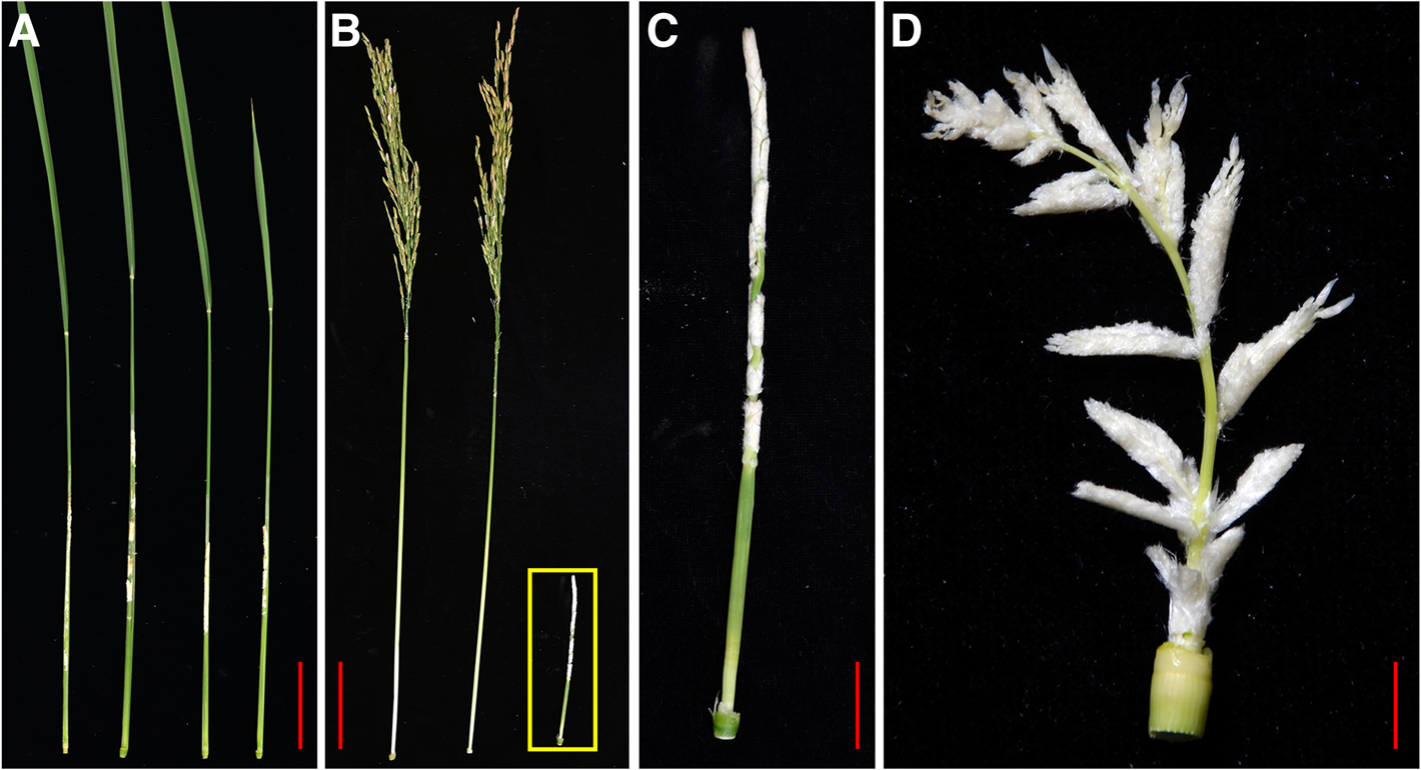
Panicle exsertion phenotypes of S142, 430, and *pds* plants at heading stage. **a** Panicle exsertion defect in *pds* plants. **b** Panicles of S142, 430, and *pds* plants at heading stage. **c** High magnification of the indicated square region in (**b**). **d** Manually stretched panicle of a *pds* plant showing dense bract hairs at heading stage. Bars = 5 cm in (**a** and **b**) and 2 cm in (**c** and **d**)

In the vegetative stage, no visible morphological difference, including plant height, tiller number, leaf number, and leaf color, was observed in the *pds* plant compared with its parents, 430 and S142 (Additional file 2: Figure S2). However, at heading stage, the panicles of 430 and S142 were fully exserted from the flag leaf sheaths, whereas most *pds* panicles appeared fully sheathed, although the panicles of a few tillers on the *pds* plants (in general, 1 per plant) were partially exserted from the sheaths (Figs. 1a and 2). These sheathed panicles lead to a significant reduction in height of *pds* plants in contrast to the parental lines (Fig. 1a-c). Further dissection showed that *pds* exhibited a shortened uppermost internode (I) and reduced panicle length (P) (Fig. 1c, d). Intriguingly, however, internode III of *pds* was significantly longer than those of its parents. The lengths of the other internodes (II, IV, V) did not significantly differ from those of their counterparts among the three genotypes (Fig. 1c, d).

**Fig. 3:**
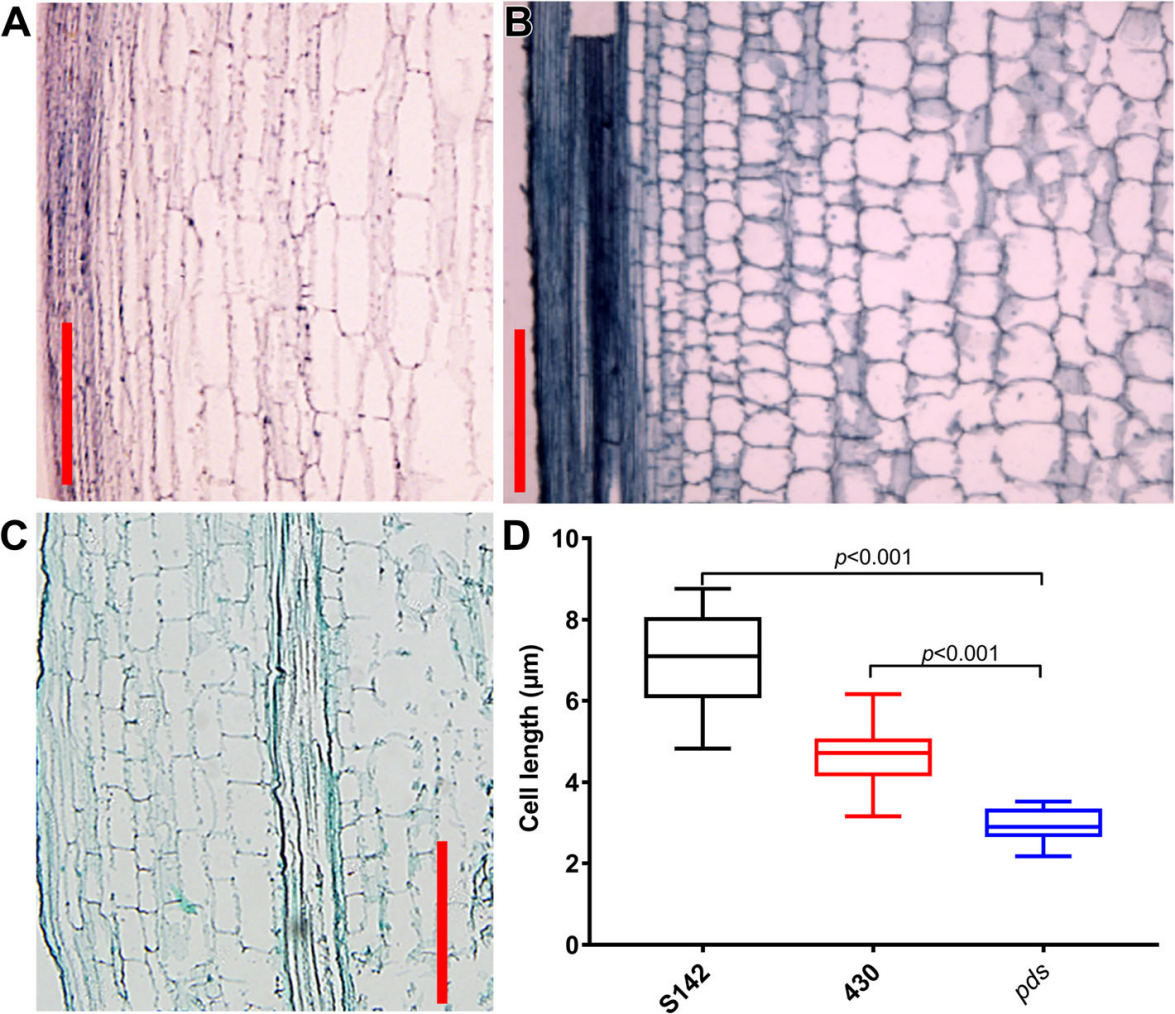
Comparison of cell lengths in the uppermost internodes of S142, 430, and *pds* plants. **a** Longitudinal section of the uppermost internode of an S142 plant. **b** Longitudinal section of the uppermost internode of a *pds* plant. **c** Longitudinal section of the uppermost internode of a 430 plant. **d** Cell lengths in the uppermost internodes of S142, 430, and *pds* plants. *P* < 0.001 in (**d**) indicates a significant difference by Student’s *t*-test when comparing the plant height of *pds* with those of its parental lines. Bars = 10 μm in (**a**, **b**, and **c**)

It has been reported that internode elongation is caused by cell division in the intercalary meristem and subsequent cell elongation in the elongation zone. Therefore, defects in the cell division and/or elongation processes could result in shortened internodes [30]. Here, longitudinal sections of the elongation zone of the uppermost internode revealed that the shortened uppermost internode in the *pds* plant was largely due to decreased cell length (Fig. 3). Moreover, significantly increased culm thickness was observed in *pds* (Fig. 1e). These results suggested that the sheathed panicle observed in the *pds* plant might be caused by the defect in cell elongation in the uppermost internode, which could lead to the shortened uppermost internode and sheathed panicle phenotype in the *pds*.

**Fig. 4:**
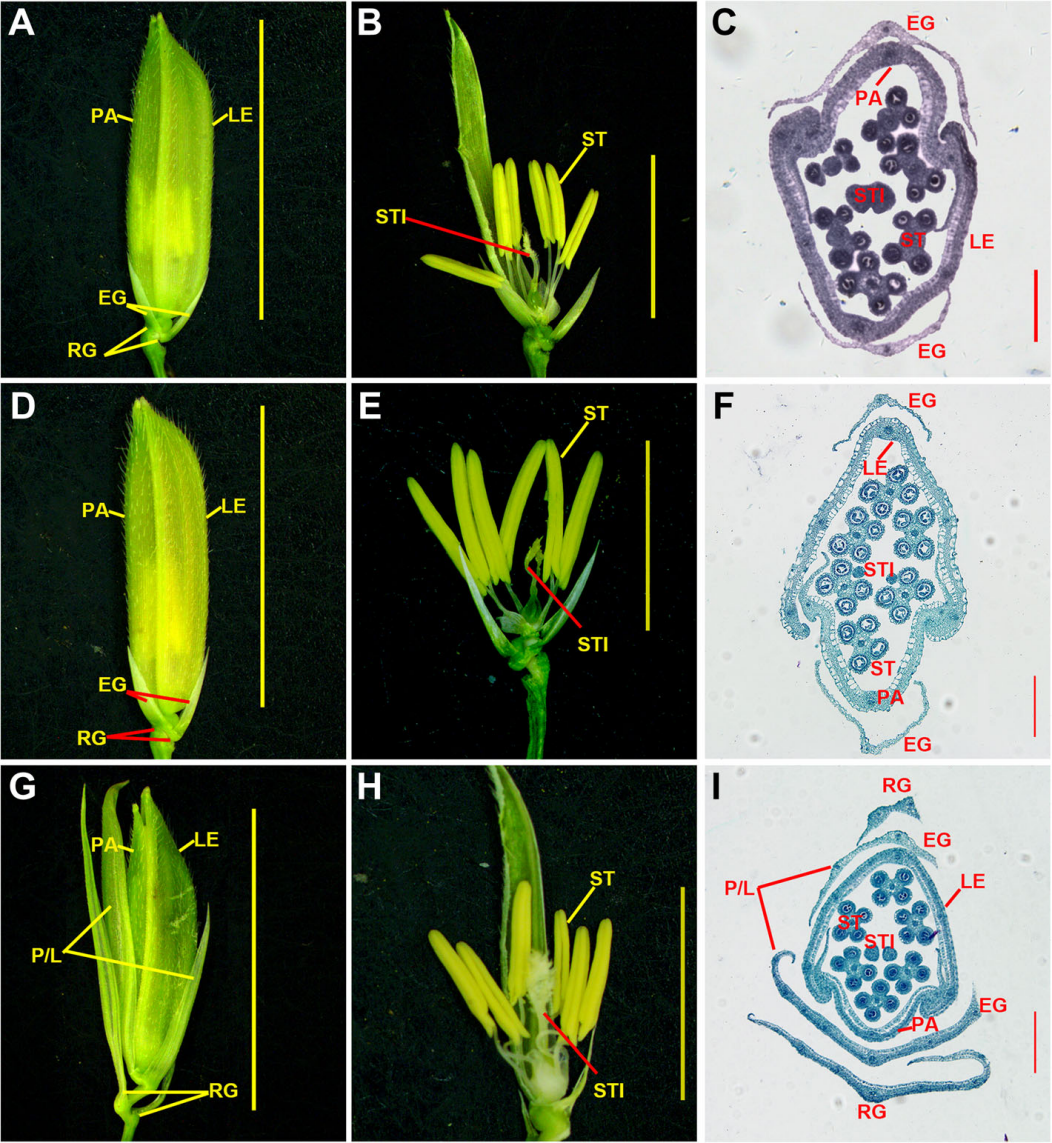
Spikelet phenotypes of S142, 430, and *pds* plants. **a** S142 spikelet. **b** S142 floret. **c** Cross-section of S142 spikelet. **d** 430 spikelet. **e** 430 floret. **f** Cross-section of 430 spikelet. **g** *pds* spikelet. **h** *pds* floret. **i** Cross-section of *pds* spikelet. PA, palea; LE, lemma; EG, empty glume; RG, rudimentary glume; ST, stamen; STI, stigma; P/LL, palea and lemma-like structure. Bars = 1 cm in (**a**, **d**, **g**), 5 mm in (**b**, **e**, **h**), and 1 mm in (**c**, **f**, **i**)

**Fig. 5:**
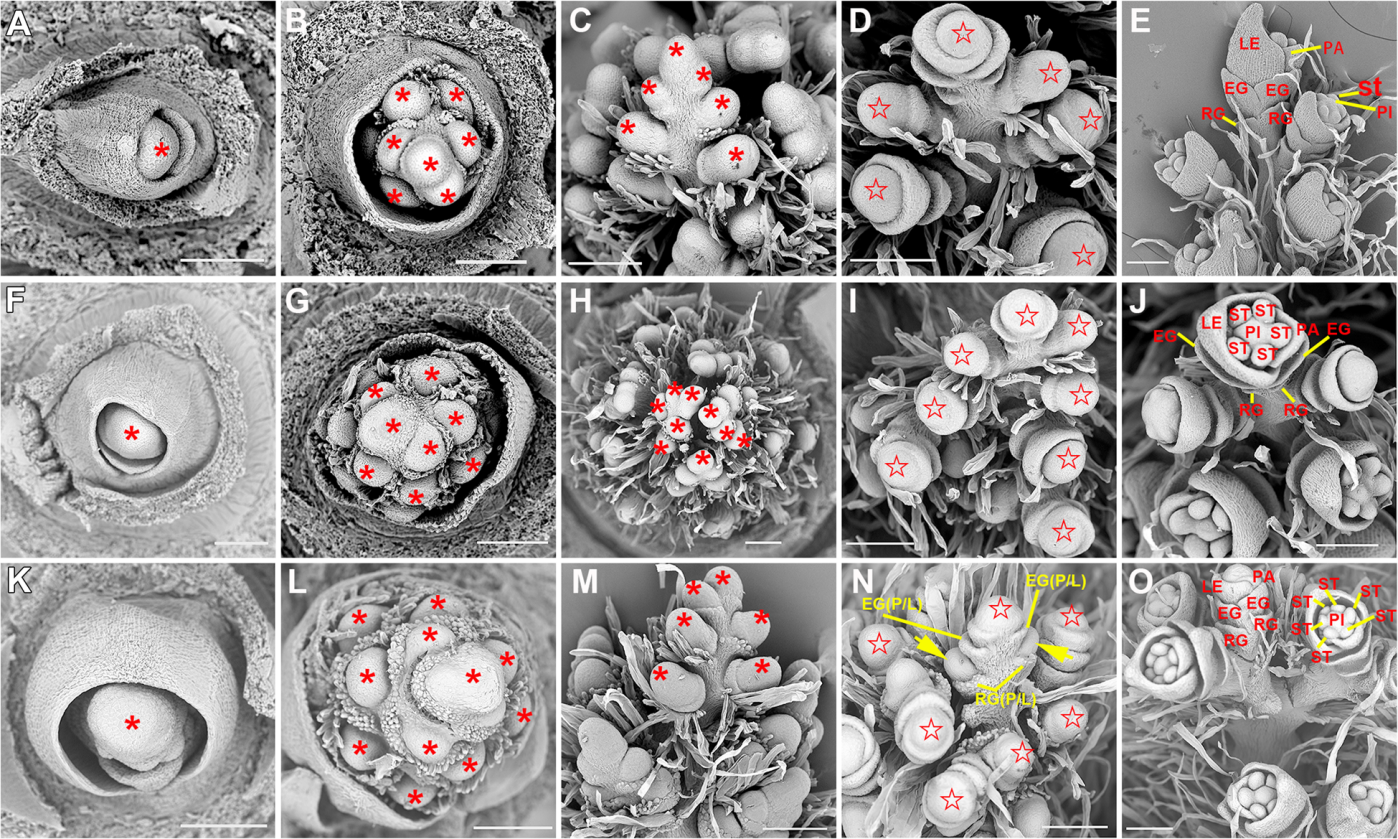
Early stages of inflorescence development in S142, 430, and *pds* plants. **a**-**e** Inflorescence development of S142. **f**-**j** Inflorescence development of 430. **k**-**o** Inflorescence development of *pds*. PA, palea; LE, lemma; EG, empty glume; RG, rudimentary glume; ST, stamen; PI, pistil, P/LL, palea and lemma-like structure. The asterisks in (**a**, **b**, **c**, **f**, **g**, **h**, **k**, **l**, **m**) represent inflorescence meristems. The pentagons in (**d**, **i**, **n**) represent floral meristems. The yellow arrows in (**n**) indicate the lateral primordia that will regenerate vegetative tillers on a small number of exserted panicles. Bars = 100 μm in all panels

**Fig. 6:**
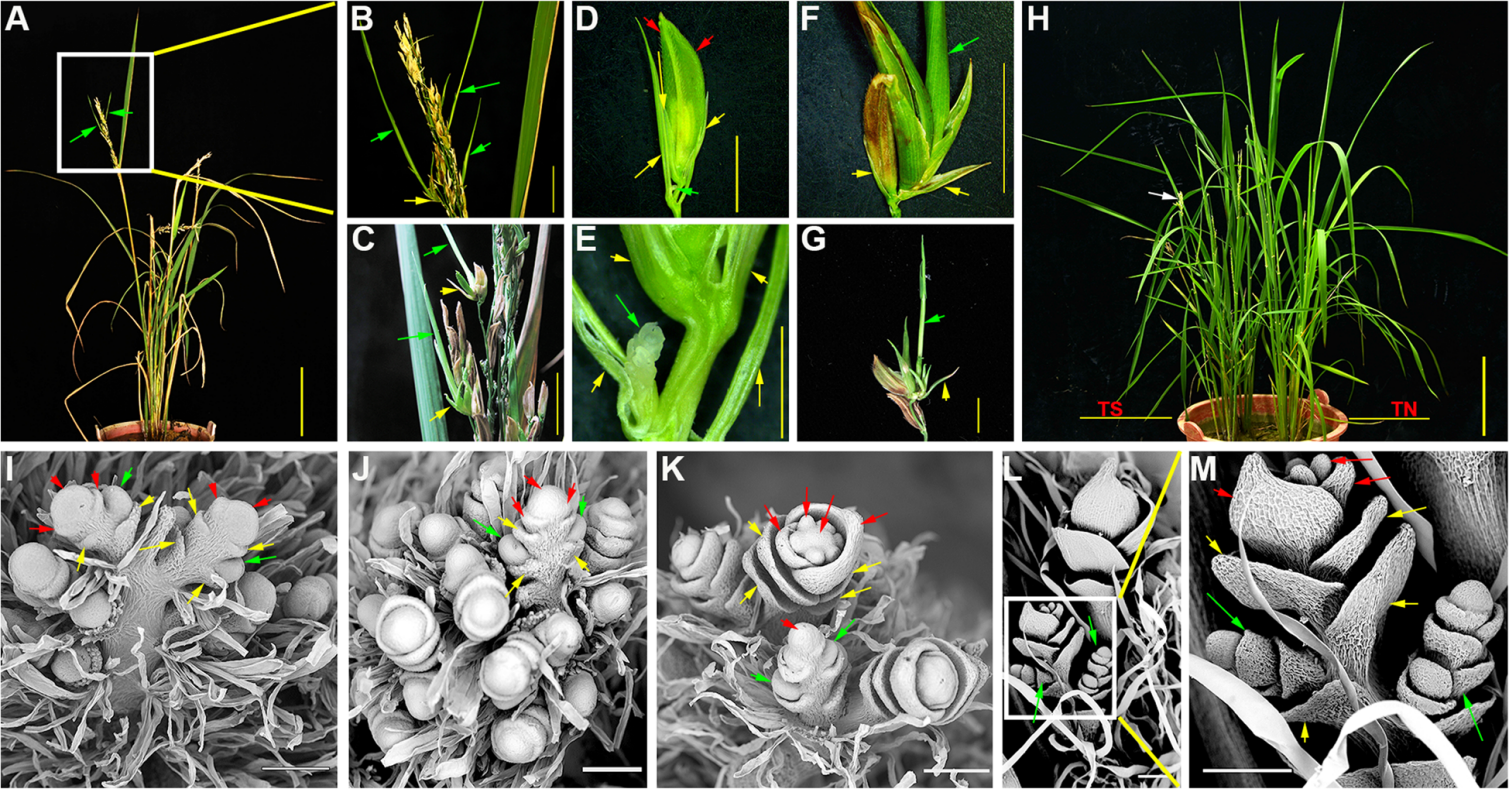
Tillering phenotype observed on a small number of exserted panicles in *pds* plants. **a** *pds* plant showing tillers on the exserted panicle. **b** High magnification of the square region in (**a**). **c** Tillers on *pds* panicle*.* **d** *pds* spikelet showing the emergence of a tiller bud at the axil of a rudimentary glume. **e** Magnification of a tiller bud at the axil of a rudimentary glume. **f**, **g** Tiller at the axil of a rudimentary glume. **h** Plants grown from cuttings of tillers on the panicle (left) and on the culm (right) of *pds*. **h**-**k** The development of *pds* spikelets showing tiller primordia emerging from the axils of the rudimentary glumes. **l** A young tiller emerging from the axil of the rudimentary glume. **m** Magnification of the square region in (**l**). The green arrows in all panels designate tillers, tiller buds, and tiller primordia emerging from the axils of rudimentary glumes of the spikelets. The yellow arrows in all panels designate the empty and rudimentary glumes of the spikelets. The red arrows in (**i**, **j**, **k**, **m**) designate the paleas, lemmas, and floral primordia of the spikelets. The white arrow in (**h**) indicates the panicle of the rice plant grown from a spikelet tiller. Bars in (**a** and **h**) = 10 cm, (**b**-**g**) = 1 cm, and (**i**-**m**) = 100 μm

**Fig. 7:**
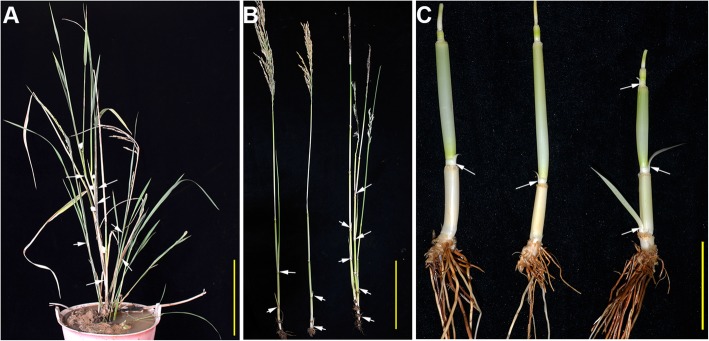
Tillering phenotype of the *pds* plant. **a** *pds* plant with higher-order tillering and a high number of tillers. **b** Tillers on the main culms of S142, 430, and *pds*. **c** Tiller buds on the stems of S142, 430, and *pds*. The white arrows in (**a**, **b**, **c**) indicate the positions of tiller formation on the rice culm. Bars in (**a** and **b**) =10 cm, (**c**) = 1 cm

**Fig. 8:**
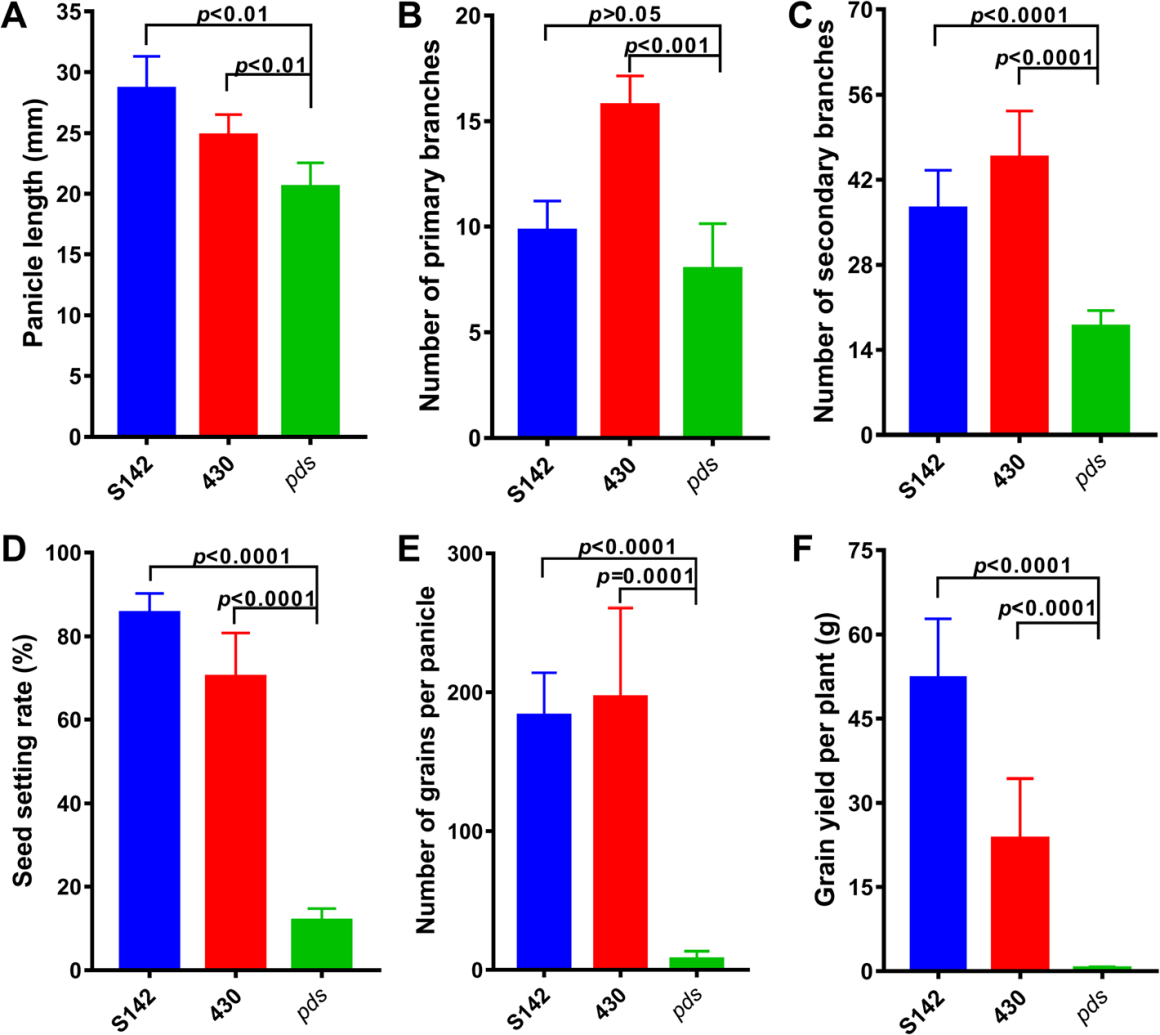
Comparison of grain yield-related traits in S142, 430, and *pds* plants*.* **a** Panicle length. **b** Number of primary branches. **c** Number of secondary branches. **d** Seed setting rate. **e** Number of grains per panicle. **f** Grain yield per plant. Error bars in (**a**-**f**) indicate the mean ± sd. *P* < 0.05 indicates a significant difference compared with the controls according to Student’s *t*-test

**Fig. 9:**
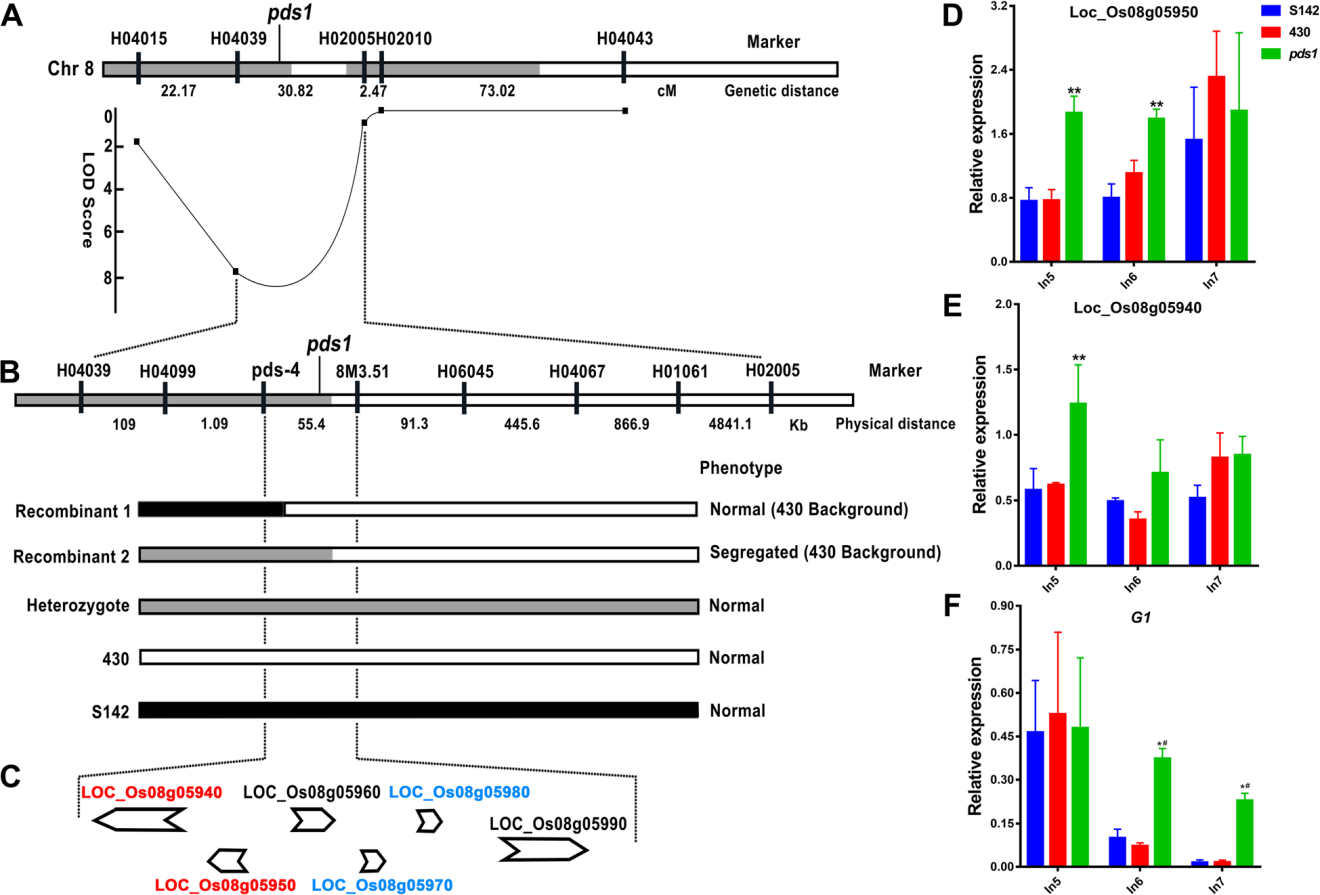
Fine mapping of *pds1* and the expression analysis of its candidate genes and spikelet development related gene at spikelet development stage In5, 6, and 7 of S142, 430, and *pds* plant. **a** Preliminary mapping of *pds1*. **b** Physical map of the *pds1* locus. Two key recombinants delimited the mapping region. **c** Putative ORFs in the mapping region. **d** The expression level of candidate gene Loc_Os08g05950. **e** The expression level of candidate gene Loc_Os08g05940. **f** The expression level of *G1* gene. Red color IDs in (**c**) indicate the candidates with significant differential expression between *pds* plant and its parents; Blue color IDs in (**c**) indicate the candidates did not show significant expression between *pds* plant and its parents; Black color IDs in (**c**) indicate the expressions of candidates were undetectable in *pds* plant and its parents

### Morphological abnormalities in *pds* spikelets

In general, a normal rice spikelet comprises two rudimentary glumes (vestigial), two small leaf-like sterile lemmas, and one fertile floret. A floret contains one pistil, six stamens, two lodicules, a lemma, and a palea (Fig. 4a-f). In the exserted panicles of *pds*, the spikelets exhibited aberrant morphology in contrast to the spikelet phenotypes of its parents, S142 and 430 (Fig. 4g-i). In the spikelet of *pds*, the two reduced sterile lemmas acquired new organ identities and thus elongated and developed into P/LL structures (Fig. 4g-i). The lengths of the sterile lemmas varied from 2.60 to 11.23 mm in the *pds* plants (Additional file 9: Table S2). More than half of the grains (60.25%) had medium-length (4.0 ≤ L ≤ 7.0 mm) sterile lemmas, while 18.55 and 21.20% of grains had short (L < 4.0 mm) and long (L > 7.0 mm) sterile lemmas, respectively (Additional file 3: Figure S3A-C). In addition, approximately 30.58% of the spikelets of the *pds* plants exhibited elongated rudimentary glumes, and their lengths ranged from 0.69 to 14.87 mm (Figs. 4g and 6d-g; Additional file 4: Figure S4G, H; Additional file 5: Figure S5E, F; Additional file 6: Figure S6; Additional file 9: Table S2). In SEM analysis, the abaxial surfaces of the sterile lemmas of S142 and 430 were smooth, and trichomes were rare except at their marginal regions. By contrast, the P/LL organs in *pds* had rough abaxial surfaces on which trichomes were formed (Additional file 4: Figure S4A-I). The surface morphology of the P/LL in *pds* was highly similar to those of the lemma and palea, suggesting that P/LLs in *pds* acquire lemma/palea identity during young spikelet development. Apart from the features mentioned above, no obvious abnormalities were detected in the course of inflorescence development or in the floral organs (Fig. 4h, i; Fig. 5). Therefore, the florets of *pds* are fertile, and seed is set at ripening stage, although the grain yield is severely decreased because of the abnormalities (Fig. 8; Additional file 7: Figure S7).

### Pseudovivipary observed in *pds* spikelets

Vivipary in flowering plants is a phenomenon in which seeds germinate and grow into plantlets while still attached to the parent plant [31]. In contrast, pseudovivipary is a process that the plantlets on the parent plant do not derive from seed germination but from buds initiated in the axils of the floral organ bracts [31, 32]. Pseudovivipary is common to grasses that grow in extreme environments [31, 32]. Strikingly, the pseudovivipary phenomenon was observed in the spikelets of *pds* plants. At heading stage, the florets of the *pds* line had normal self-pollination and continued normal embryo and grain development; however, at the same time, the development of plantlets (tillers) from the axils of the elongated rudimentary glumes was occasionally detected in the spikelets of *pds* panicles (commonly, 2–3 per panicle; ~ 20% of exserted panicles) (Fig. 6a-g). We called these plantlets panicle tillers (PTs). PTs had the characteristics of normal juvenile plants; they could produce additional tillers and continue their normal vegetative and reproductive growth, similar to shoot tillers, when transplanted into paddy soil (Fig. 6g, h). In line with these morphological observations, SEM analysis revealed that PT primordia were ectopically initiated in the axils of the rudimentary glumes (bracts) during early spikelet development in *pds* (Fig. 5d, i, n; Fig. 6i-m). However, not all *pds* spikelets formed ectopic primordia. The abnormal spikelets formed either one primordium (Fig. 5n; Fig. 6i, l, m) or two in the axils of a pair of rudimentary glumes (Fig. 5 n and Fig. 6 j, m). These results suggested that the AMs in the axils of the rudimentary glumes did not degenerate during normal rice spikelet development and were instead suppressed by one or more unknown regulators. Thus, when this suppression was removed, the AMs acquired axillary tiller primordium identity and developed into PTs.

### Ectopic development of upper node tillers in the *pds* plants

During the vegetative development of rice, tiller primordia are initiated during the time of leaf primordium formation, and then, the tiller primordia develop into tiller buds [1]. However, in subsequent stages of development, only the tiller buds on the nodes adjacent to unelongated internodes on the rice culm develop into tillers, whereas the tiller buds on the upper, elongated internodes become dormant [1]. In *pds*, the normal tillering pattern of rice plants was observed in the vegetative stage, similar to that of the parents (Additional file 2: Figure S2A), although later events suggested that the upper node tiller buds had not entered dormancy (Fig. 7c). Growth of the tiller buds on the upper nodes was observed in sheathed-panicle *pds* plants after heading stage (Fig. 7a, b). The sheathed, aborted panicle phenotype of *pds* plants is unfavorable for reproduction. To ameliorate such deleterious effects, plants have evolved certain strategies to survive harsh environments. Our results suggested that the development of the upper-node tillers might be a compensatory strategy allowing rice plants to propagate in unfavorable circumstances, although no panicles were found exserted from the upper nodes of the mature tillers, or genetic incompatibility led to the disorder of the development pattern of rice.

### Reduced grain yield in the *pds* line

At ripening stage, up to 80% of the panicles of the *pds* plants were sheathed, and the fully exserted panicles were smaller and shorter than those of the parental lines (Fig. 1a, c). The panicle length of *pds* was 29.04% shorter than that of S142 and 18.05% shorter than that of 430 (Fig. 8a; Additional file 9: Table S3). The primary branch number of *pds* was significantly lower than that of 430, and the secondary branch number was significantly decreased compared with those of S142 (by 32.35%) and 430 (by 47.13%), respectively (Fig. 8b, c; Additional file 9: Table S3). Moreover, compared with those of its parents, the seed setting rate, number of grains per panicle, and grain yield per plant were dramatically reduced in *pds* plants (Fig. 8d, e, f; Additional file 9: Table S3). These results indicated that the grain yield of *pds* was significantly affected because of its abnormalities.

### Genetic analysis of *pds* and fine mapping of *pds1*

To examine the genetic basis of the phenotypes characterized in the *pds* line, reciprocal crosses between *pds* and its two parents, S142 and 430, were performed to obtain genetic materials for genetic linkage analysis. The F_1_ plants of every cross (*pds*×S142, *pds*× 430) exhibited normal phenotypes, similar to those of the parental lines. In the case of cross between *pds* and 430, 88 rice plants exhibited normal phenotype, and 34 showed abnormal phenotype in a 122 individual F_2_ populations (normal: aberrant≈3:1, χ^2^ = 0.546 < χ^2^_0.05_ = 3.84) in 430 background. Likewise, in a F_2_ population with 93 individuals, 78 plants exhibited normal phenotype and 15 showed abnormal phenotype (normal: aberrant≈3:1, χ^2^ = 0.146 < χ^2^_0.05_ = 3.84) in S142 background (Table 1). The results indicated that a S142-derived recessive locus, genetically interacted with another recessive locus from 430, involved in controlling the establishment of abnormal phenotype. We designated this two loci *pds1* and *pds2*, respectively. The pyramiding of both *pds* loci resulted in the severe defect in the development and exsertion of rice panicle, suggesting the genetic incompatibility in the hybrid. Such incompatibility might lead to reproductive isolation or hybrid weakness in the evolutionary history of rice. Here, to identify the *pds* loci underlying the abnormal *pds* phenotype, the polymorphic markers between the two parental lines were screened for linkage analysis. One hundred seventeen polymorphic markers that were evenly distributed on the 12 chromosomes of rice were obtained. For *pds1* locus, a linkage analysis revealed that the polymorphic markers were highly linked to the *pds* phenotype (Fig. 9a). To further fine-map *pds1*, a large population and high-resolution polymorphic markers were developed to screen the recombinant events that occurred in the interval between markers. Using a 3900-individual F_2_ population, two important recombinants were identified, and *pds1* was located to a 55.4-kb interval (Fig. 9b). The region contained six RGAP-annotated putative ORFs (Fig. 9c). Of these, LOC_Os08g05940 and LOC_Os08g05990 encode retrotransposon proteins. LOC_Os08g05950, LOC_Os08g05960, LOC_Os08g05970, and LOC_Os08g05980 are putative expression proteins. For *pds2* locus, however, it was only got preliminary mapped recently and do not publish in this article.

**Table 1:** Genetic analysis of *pds* phenotype

Putative locus	Donor parent	Cross	F_1_ phenotype	F_2_ phenotype		χ^2^	χ^2^_0.05_	3:1 ratio
				Normal	Abnormal			
*pds1*	S142	*pds* × 430	Normal	88	34	0.546	3.84	Fitted
*pds2*	430	*pds* × S142	Normal	78	15	0.146	3.84	Fitted

Notes:χ^2^ indicates chi-square test value; χ^2^_0.05_ indicates chi-square test value at *p*-value = 0.05

### Expression analysis of the candidate genes for *pds1* and the genes related to spikelet development

According to the previously defined inflorescence development staging [3], the development of spikelets in rice occurs during stages In6–7. During these two stages, pairs of rudimentary glumes, sterile lemmas, paleas, and lemmas differentiate sequentially. The involvement of the *G1* and *lhs1* (*OsMADS1*) genes in specifying sterile lemma development has been reported in previous studies [4, 8]. Furthermore, double mutation in *OsMADS1* and *OsMADS15* results in the transformation of rice spikelets/flowers into juvenile plantlets. To determine the probable gene underlying *pds1* locus among those 6 candidate genes in the mapping interval, and the relationships between the *pds* loci and the characterized spikelet development related genes at the transcriptional level, qPCR was performed to examine the expression of related genes at different stages of spikelet development in *pds* and its parental lines. The expression patterns of candidate gene Loc_Os08g05950 at stage In5 and In6 of *pds* plant were significantly up-regulated by comparing with those in S142 and 430. The expression profile of another gene Loc_Os08g05940 only showed significantly up-regulated in stage In5 (Fig. 9d, e). For the other 4 candidates, the expression levels of Loc_Os08g05970 and Loc_Os08g05980 at either stage of *pds* plant were observed no significantly difference by contrasting to S142 and 430, whereas, the expression of Loc_Os08g05960 and Loc_Os08g05990 were undetectable in the test samples (Additional file 8: Figure S8 A, B). The results indicated Loc_Os08g05950 is a most possible candidate for *pds1*. However, little is known about the function of this candidate gene from recent literatures.

Regarding to the spikelet development related genes, the expression patterns of the *G1* gene at the In6 and In7 stages in *pds* were significantly different from those in S142 and 430 (Fig. 9f). In contrast, the expression patterns of *OsMADS1* and *OsMADS15* did not differ at the In6 and In7 stages in the three genotypes, although their expression was higher at stage In5 in *pds* than in its parents (Additional file 8: S8C, D). These results suggested that *G1* might be involved in the genetic mechanisms underlying the *pds* abnormal phenotype observed in this study.

## Discussion

In this study, we characterized a rice RIL, *pds* with many developmental events being disturbed in all life stages. Its abnormal phenotype includes fully sheathed panicles, elongated sterile lemmas, the development of tillers in the axils of the rudimentary glumes, and ectopic tillers produced on the upper nodes of the main culm. Our results suggested that *pds1*, one of the major loci underlying *pds* phenotype, might regulate the activity of AMs in response to the disturbance of the reproductive program caused by genetic incompatibility.

### Genetically incompatible recessive loci cause abnormal phenotypes in rice

The *pds* line was derived from a cross between the lines S142 and 430, which exhibited normal plant architecture and normal spikelet development. Based on a genetic analysis, the recessive locus *pds1*, genetically interacted with *pds2*, was demonstrated to control the abnormalities of the *pds* phenotype. Although the *pds1* allele was derived from S142, the effects of a single recessive locus of *pds1* and *pds2*, the one yet to be identified, were masked by *PDS2* and *PDS1* in both S142 and 430, respectively, implying that dominant epistatic effects are exerted on these recessive loci. When the recessive loci were pyramided by the cross, these masks were removed, and the abnormal phenotypes were appeared in the hybrids. These results indicated that *pds1* and its interactor are genetically incompatible in the hybrids. Cases of genetic incompatibility causing hybrid weakness in crops, at both the interspecific and intraspecific levels, have been reported in several studies [26–29]. Recently, Chen et al. [29] discovered a novel type of interspecific hybrid weakness in rice. In this case, two incompatible loci, *Hwi1* and *Hwi2*, which were isolated in a CSSL line derived from a cross between the *indica* rice Teqing (*Oryza sativa* L.) and a common wild rice (*Oryza rufipogon*) strain, caused impaired root formation and dwarfed plant stature [29]. That case of hybrid weakness was induced by the interaction between the *Hwi2* and *Hwi1* alleles, which were pyramided by the cross between Teqing (*Hwi2*) and wild rice (*Hwi1*) in the Teqing background. For the interpretation of the genetic incompatibility phenomenon, the Dobzhansky-Muller (DM) model postulates that genetic incompatibility is caused by deleterious interactions between different alleles that arose in divergent lineages during evolutionary history [33, 34]. Therefore, we speculated that either *pds1* and its interactor might evolve from two independent lineages of *Oryza*, resulting in incompatibility after fertilization, or incompatible mutations arose in the sequences of *pds1* and its interactor during domestication respectively, resulting in hybrid weakness in the hybrids of two rice varieties. To confirm this hypothesis, further isolation and characterization of *pds1* and *pds2* would facilitate a better understanding of the origins of these loci via molecular phylogenetic analysis.

### Bract AMs are maintained throughout rice development

During postembryonic growth, AMs sequentially acquire lateral organ identity and initiate lateral branch or flower development in the vegetative and reproductive phases, respectively. In rice, under normal developmental regulation, the tiller buds on the upper nodes become dormant, and the panicles develop with normal morphology. When normal regulation is disturbed, the specification of the axillary buds or meristems become uncontrolled, and the ectopic organs may develop in the axils of the bracts (leaves and glumes). Several genes involved in the maintenance of AM fate have been identified by studying mutants with developmental defects in rice. The *Asp1* mutant exhibits numerous abnormal phenotypes, including disorganized branching, aberrant spikelet morphology, and leaf phyllotaxy alteration. *ASP1* encodes a transcriptional co-suppressor that is related to TOPLESS (TPL) in *Arabidopsis*. The mutant also showed de-suppression of axillary growth in the vegetative phase, which resulted in the development of tiller buds in the axils of the upper nodes of the rice main culm [23]. Furthermore, intriguingly, a study of the *phoenix* (*pho*) mutant showed the ectopic development of vegetative AMs or primordia in the axils of the spikelet bracts (rudimentary and sterile lemmas), namely pseudovivipary. In fact, the *pho* mutant was shown to be a double mutant containing a mendelian mutation in *DEP* (*OsMADS15*) and a non-mendelian mutation in the *AFO* gene (*OsMADS1*). This mutant generates new plantlets in its spikelet termini in place of the normal floral organs, suggesting that the AMs in the axils of the lemmas acquired tiller bud identity and developed as plantlets on the panicle [9]. This phenomenon also can be observed in the overexpression of *OsLEC1* in rice [11]. In addition, lateral florets are ectopically generated in the axils of the sterile lemmas in *lf1* mutant spikelets [10]. In the present study, another case of pseudovivipary was found in the rice *pds* line, namely, the ectopic development of tillers in the axils of the rudimentary glumes of spikelets and on the upper nodes of the main culm. All the cases discussed above suggest that *PDS1* and at least one additional locus genetically interact to control the determinacy of AMs, including those initiated in both the vegetative phase and the reproductive phase. Of particular interest, in terms of the fate of the SPMs, it has been reported that SPM indeterminacy is lost upon their transition from branch meristems in rice [5, 8, 35]. However, previous studies and our results suggested that the indeterminacy of spikelet AMs might never be lost during spikelet development but only suppressed under normal conditions by certain regulatory genes.

### Rudimentary glumes and sterile lemmas have different origins

The glumes are unique structures in grasses [36]. More than 200 years ago, Goethe proposed that the floral organs are modified leaves. According to comparative morphological study, the first pair of bracts (rudimentary glumes) differentiated from the SPMs resemble the leaf primordia produced in the vegetative phase, based on their position. This result supports the hypothesis that floral organs are modified leaves. The subsequent pair of small glumes (sterile lemmas) subtend the floral organs and exhibit remarkable morphological differences from the rudimentary glumes. In particular, the surface and abaxial cell shape of the rudimentary glumes are completely different from those of the sterile lemmas, implying that these organs have different origins and different identities [37]. Two popular points have been proposed to interpret their origins, although the matter remains controversial. The first widely accepted point is that the rudimentary glumes are bracts subtending the spikelet, whereas sterile lemmas are part of the spikelet [1, 38, 39]. An alternative proposal suggests that the sterile lemmas are two reduced lateral florets, while the rudimentary glumes are the equivalents of the glumes found in other grass species [39]. In this study, the tiller buds produced in the axils of the rudimentary glumes suggest that the origin and identity of the rudimentary glumes are similar to those of the leaves produced in the vegetative phase, which subtend the shoot tiller buds. These results, taken together with an observation from the *lf1* mutant, in which lateral florets were formed in the axils of the sterile lemmas [10], support the notion that the sterile lemmas evolved from two reduced lateral florets, giving them a different origin and identity from the rudimentary glumes.

### *PDS1* might maintain the normal sexual organ development and reproduction process with its partner in rice

Life-history strategies combining sexual and asexual reproduction can result in stable population structures under variable environmental conditions, because the two modes of reproduction are successful in different circumstances [40, 41]. In flowering plants, although flowering is typically an irreversible event, reversion from floral to vegetative growth occurs frequently in nature [9]. Pseudovivipary, which floral organs are replaced by bulbils or plantlets, is a reversion event that can occur when plants encounter extreme circumstances, providing an asexual means for many monocots to reproduce and disperse [9, 41–43]. However, in the present study, stable pseudovivipary was observed in the *pds* line, indicating that this phenotype is not associated with environmental factors. Although the molecular basis of pseudovivipary remains unknown, we deduced that the process is controlled by both genetic and environmental factors. Thus, we proposed that the hybrid weakness observed in *pds* line, including the fully sheathed panicle, aborted young panicle, and aberrant development of spikelets due to the genetic interaction between *pds1* and *pds2* resulting in the disorder of development events and a failure of sexual reproduction in rice. This failure causes a reversion from sexual to asexual reproduction and leads to the development of plantlets/tillers in the axils of the rudimentary glumes and on the upper nodes of the main culm. Therefore, *PDS1* and its interactor might act as co-suppressors to inhibit the disorder of development pattern and maintain the normal sexual organ development and reproduction process in rice. Moreover, pseudovivipary might be a compensatory mechanism allowing plants to propagate in harsh circumstances, including internally and externally unfavorable conditions, or the genetic incompatibility leading to the disorder of the development pattern of rice.

## Conclusion

In summary, our study observed genetic incompatibility or hybrid weakness caused by a genetic interaction between loci derived from different rice varieties carrying incompatible *pds* loci. The unraveling of the genetic mechanisms and genetic relationships among these loci underlying rice inflorescence development is of great significance for rice yield improvement, and further isolation and characterization of *pds* loci is warranted. Additional study will shed new light on the mechanisms of grass inflorescence development.

## Methods

### Plant materials

All the plant materials used in this study were grown in the experimental field and deposited in the Germplasm repository of Guangxi University complying with legislation of China. The RIL *pds* was provided by Zhongquan Cai, a co-worker who works on rice breeding in our group. It was found in a rice breeding program which aimed at breeding a novel restorer line for ‘three-line’ hybrid system by crossing S142 with 430. The two parental lines are *indica* restorers that were previously bred as intermediate breeding materials by Zhongquan Cai (Agriculature College, Guangxi University). S142 exhibits more tiller number but with small panicles, whereas, 430 exhibits stronger culm, bigger panicles and lower tiller number. The RIL *pds* was found from one of their F_2:3_ family. *Pds* plants exhibit severe panicle exsertion defects. However, a small number of panicles with aberrant spikelets half-emerged from their sheaths, and the 4 whorls of these florets developed normally (Fig. 4b, e, h). From these plants, *pds* seeds were collected and grown for 6 consecutive generations to confirm the heritability and homozygosity of the phenotype. This *pds* line was backcrossed at the sixth generation with its parents. The F_1_ plants were self-pollinated to obtain the F_2_ population to perform genetic analysis to determine genetic basis of aberrant phenotype, and for *pds* loci fine mapping. All field trials were performed in the experimental field at the campus of Guangxi University in compliance with the laws of China.

### Microscopic examination

The fresh spikelets of S142, 430, and *pds* plants were collected and fixed in FAA solution (formalin: glacial acetic acid:70% ethanol; 1:1:18). The specimens were subjected to vacuum for approximately 15 min to ensure that the materials were completely immersed in the FAA solution and then fixed at 4 °C overnight. After fixation, the specimens were dehydrated with a gradient ethanol series (30, 50, 70, 85, 95, 100, and 100%). Each step of the dehydration was incubated for 1 h. The dehydrated specimens were cleared/infiltrated in xylene and then embedded in Surgipath Paraplast (Leica).

The specimens were sectioned into 8-μm thick sections using a microtome (Leica RM2235). The ribboned sections were placed on glass slides and floated in milli-Q water on a hot plate set at 40 °C for 30 min. The stretched sections were then deparaffinated with serial xylene and rehydrated with a gradient ethanol series (100, 95, 85, 70, 50, and 30%). The sections were stained using 1% safranin O and 0.5% Fast Green and then dehydrated and cleared. The sections were finally covered with neutral resins at room temperature for 48 h and then examined by optical microscopy (Olympus BX51, Japan). The cell length was measured using ImageJ software. Three biological replicates were examined to obtain the final results.

### SEM analysis

The phenotypes of young panicle development in S142, 430, and *pds* were examined using scanning electron microscopy (SEM). The young panicles of different development stages were dissected and fixed overnight at 4 °C in FAA (formalin: glacial acetic acid:70% ethanol; 1:1:18) and then dehydrated with a gradient ethanol series. The samples were then TBA (tert-butyl alcohol)-dried, mounted, and sputter-coated with platinum. The samples were observed and photographed using a scanning electron microscope (F16502, Phenom Ltd., Netherlands).

### Fine mapping of loci controlling the *pds* phenotype

The preliminary mapping of the loci for the *pds* phenotype was performed using the F_2_ populations generated by backcrossing between *pds* and each of the parents. Genome-wide polymorphic markers between S142 and 430 were identified using SSR and IN/DEL markers. SSRs were obtained from the Gramene database (http://www.gramene.org/). The IN/DEL markers were developed using the genomic DNA sequences of Nipponbare and 9311 as references. The genotyping of the polymorphic markers of the respective F_2_ populations was conducted using PCR. QTL IciMapping 4.1 [44] was used to construct a genetic linkage map and perform genetic locus analysis based on the genotypes and phenotypes of the mapping populations following the software manual. In QTL analysis, the statistical method Inclusive Composite Interval Mapping (ICIM-ADD, ICIM-EPI) was used for genome-wide identification of loci associated with the *pds* phenotype. A threshold of LOD ≥ 3.0 was used to indicate the significant main effect QTL (*P* ≤ 0.001). For fine mapping of the identified loci, a large F_2_ population was planted, and new IN/DEL markers were developed to identify recombinants in the target region of each putative *pds* locus. The phenotypes of the recombinant plants were determined by F_3_ progeny testing. Linkage analysis was performed to narrow down the *pds* loci into small intervals.

### Quantitative real-time reverse transcription PCR analysis

The young panicles (stages In5–7) of rice plants were collected and frozen immediately in liquid nitrogen for total RNA extraction (three biological replicates per sample). Total RNA was isolated using TRIzol reagent following the manufacturer’s instructions. cDNA synthesis was performed by reverse transcription (RT) with the Thermo Scientific RevertAid First Strand cDNA Synthesis Kit (Cat# K1622) according to the manufacturer’s protocol. The sequences of the genes that control rice inflorescence development were downloaded from the Rice Genome Annotation Project (RGAP) [45]. The primers for related genes were obtained from qPrimerDB (https://biodb.swu.edu.cn/qprimerdb/) (Additional file 9: Table S1) [46]. qPCR was performed using a Roche LightCycler 480 Real-Time PCR System in 10 μL reactions with the SYBR Green PCR Master Mix kit (Bio-Rad, USA) to detect the relative expression of these genes following the manufacturer’s protocol. The relative expression of each gene was calculated according to the 2^-△△CT^ method [47]. The *Actin* gene (LOC_Os11g06390) was used as an endogenous reference for qPCR.

## Additional files

Additional file 1: **Figure S1.** Schematic representation of rice plant, panicle architecture, and spikelet development. (A) Rice plant showing the basal and upper tillering nodes. (B, C) Structures of the young and mature spikelets, respectively. (D) Mature rice panicle. The red dots and blue dots in (A) represent upper and basal tillering nodes, respectively. In (B, C), LE, lemma; PA, palea; OSL, outer sterile lemma; ISL, inner sterile lemma; ORG, outer rudimentary glume; IRG, inner rudimentary glume. In (D), TS, terminal spikelet; LS, terminal spikelet; SB, secondary branch; PB, primary branch; DP, degenerated point. (TIF 4615 kb)

Additional file 2: **Figure S2.** Comparison of the plant architecture of S142, 430, and *pds* at the vegetative stage. (A) Morphology of S142, 430, and *pds*. (B) Plant height of S142, 430, and *pds*. (C) Tiller number of S142, 430, and *pds*. (D) Leaf number of S142, 430, and *pds*. (TIF 24788 kb)

Additional file 3: **Figure S3.** Phenotypes of the elongated sterile lemmas in the spikelets of the *pds* line. (A) Percentages of three types of elongated sterile lemma. (B) Comparison of the sterile lemma to grain length ratio of S142, 430, and *pds*, showing the significant elongation of sterile lemmas in *pds.* (C) Comparison of sterile lemma lengths in S142, 430, and *pds*, showing the significant elongation of sterile lemmas in *pds.* Error bars in (B and C) indicate the mean ± sd; ^∗∗∗^Significant difference at *P* < 0.01 compared with the controls according to Student’s *t*-test in (B and C). (TIF 3150 kb)

Additional file 4: **Figure S4.** Scanning electron microscopy (SEM) analysis of the development of the empty glume and rudimentary glume in the spikelets of S142, 430, and *pds* plants. (A) Empty glume and rudimentary glume of S142. (B) Epidermal surface of the empty glume of S142. (C) Epidermal surface of the palea and lemma of S142. (D) Empty glume and rudimentary glume of 430. (E) Epidermal surface of the empty glume of 430. (F) Epidermal surface of the palea and lemma of 430. (G) Empty glume and rudimentary glume of *pds*. (H) Epidermal surface of the empty glume of *pds*. (I) Epidermal surface of the palea/lemma-like structures of *pds*. PA, palea; LE, lemma; EG, empty glume; RG, rudimentary glume; ST, stamen; STI, stigma; P/LL, palea/lemma-like structure. The yellow arrows in (E, F, G, I) designate the trichomes on the surfaces of the spikelet organs. Bars = 100 μm in all panels. (TIF 16971 kb)

Additional file 5: **Figure S5.** Morphological phenotypes of the spikelets of S142, 430, and *pds* plants, showing the alteration of the rudimentary glumes in the *pds* line. (A, C, E) Secondary rachis branches of S142, 430, and *pds* plants with mature spikelets, respectively. (B, D, F) Magnifications of the boxed regions in (A, C, E), respectively. The yellow arrows in (B, D, F) show the rudimentary glumes of the spikelets. The red asterisk in (F) shows an elongated pedicel. Bars =1 mm in (A-F). (TIF 5364 kb)

Additional file 6: **Figure S6.** Morphology of *pds* spikelets, showing the different types of elongation of the sterile lemmas, rudimentary glumes, and pedicels. (A) Spikelet with two nearly equal rudimentary glumes and two different lengths of sterile lemmas. (B) Spikelet with two nearly equal sterile lemmas and one elongated rudimentary glume. (C) Spikelet with two different lengths of rudimentary glumes and two different sterile lemmas. (D) Spikelet with two different lengths of rudimentary glumes, two different lengths of sterile lemmas and plantlets in the axils of the rudimentary glumes. The yellow and red arrows in (A-D) represent the rudimentary glumes and sterile lemmas, respectively. The light blue arrows in (D) show the plantlets in the axils of the rudimentary glumes. Bars = 1 mm in (A-D). (TIF 2248 kb)

Additional file 7: **Figure S7.** Seed morphology of S142, 430, and *pds* plants. (A) Seeds of S142, 430, and *pds*. (B) Empty glumes of the seeds of S142, 430, and *pds*. Bars = 1 cm in (A), 3 mm in (B). (TIF 10152 kb)

Additional file 8: **Figure S8.** The expression pattern analysis of *pds1* candidate genes and spikelet development related genes at spikelet development stage In5, 6, and 7 of S142, 430, and *pds* plant. (A) The expression level of candidate gene Loc_Os08g05970. (B) The expression level of candidate gene Loc_Os08g05980. (C) The expression level of spikelet development related gene *OsMADS1*. (D) The expression level of spikelet development related gene *OsMADS15*. (TIF 1983 kb)

Additional file 9: **Table S1.** The primer sequences of related genes using for qPCR. **Table S2.** The descriptive statistical analysis of the length of sterile lemma and rudimentary glume. **Table S3.** Grain yield related traits of S142, 430, and *pds* rice plants. (DOCX 23 kb)

## Abbreviations

AM(s): Axillary meristem(s)
DM: Dobzhansky-Muller
FM: Floral meristem
IM: Inflorescence meristem
IN/DEL: Insertion/deletion
P/LL: Palea/lemma like
*pds*: Panicle exsertion defect and aberrant spikelet
PTs: Panicle tillers
RGAP: Rice Genome Annotation Project
SAM: Shoot apical meristem
SPM: Spikelet meristem
TBA: Tert-butyl alcohol
