# Supplementary figures and images for: The rice pds1 locus genetically interacts with partner to cause panicle exsertion defects and ectopic tillers in spikelets
Source: BMC Plant Biol. 2019 May 15;19:200. doi: 10.1186/s12870-019-1805-z (PMC6521401; doi:10.1186/s12870-019-1805-z)

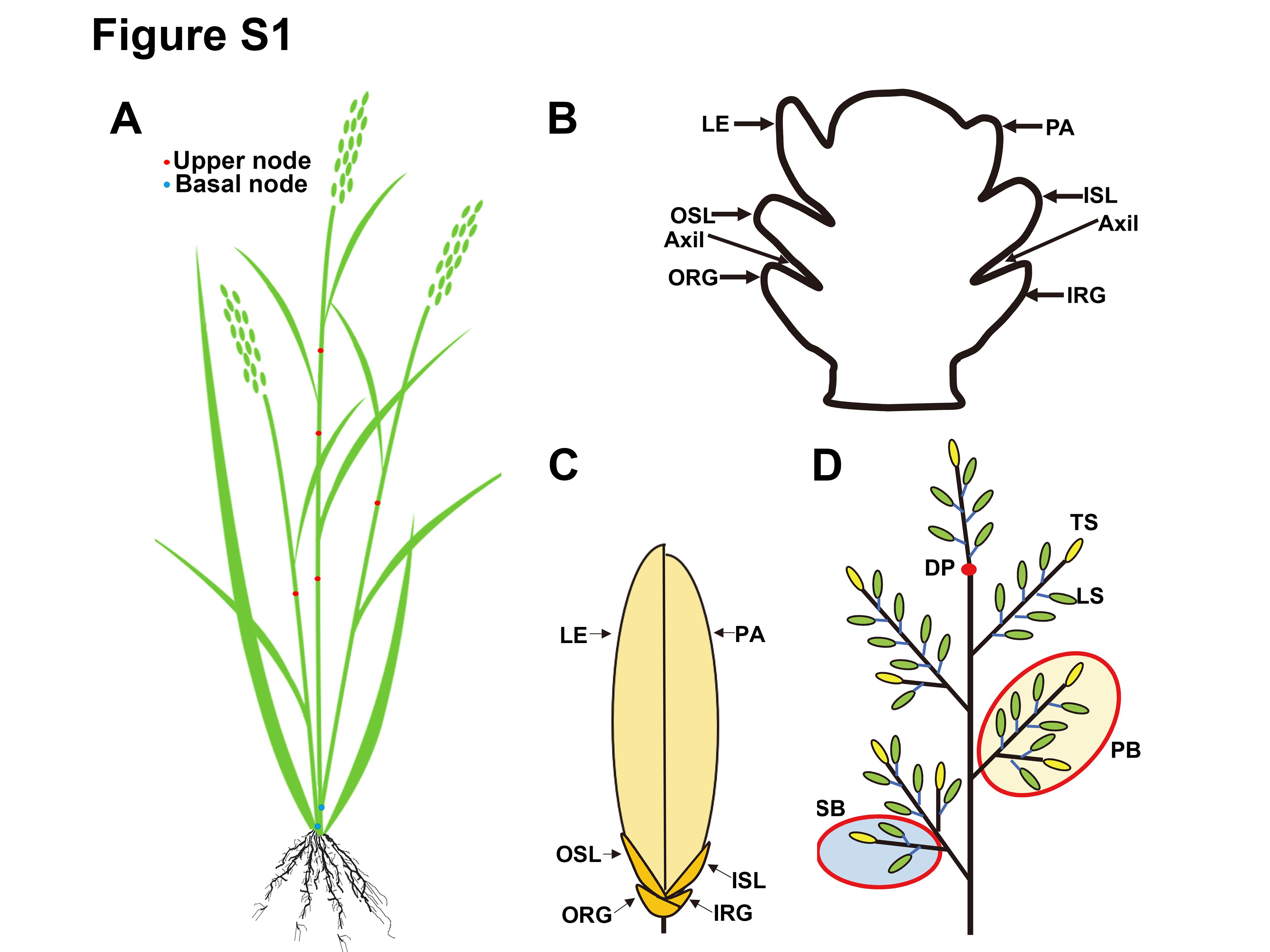

Supplement: Supplementary file 1 — Figure S1. Schematic representation of rice plant, panicle architecture, and spikelet development. (A) Rice plant showing the basal and upper tillering nodes. (B, C) Structures of the young and mature spikelets, respectively. (D) Mature rice panicle. The red dots and blue dots in (A) represent upper and basal tillering nodes, respectively. In (B, C), LE, lemma; PA, palea; OSL, outer sterile lemma; ISL, inner sterile lemma; ORG, outer rudimentary glume; IRG, inner rudimentary glume. In (D), TS, terminal spikelet; LS, terminal spikelet; SB, secondary branch; PB, primary branch; DP, degenerated point. (TIF 4615 kb) [file 12870_2019_1805_MOESM1_ESM.tif]

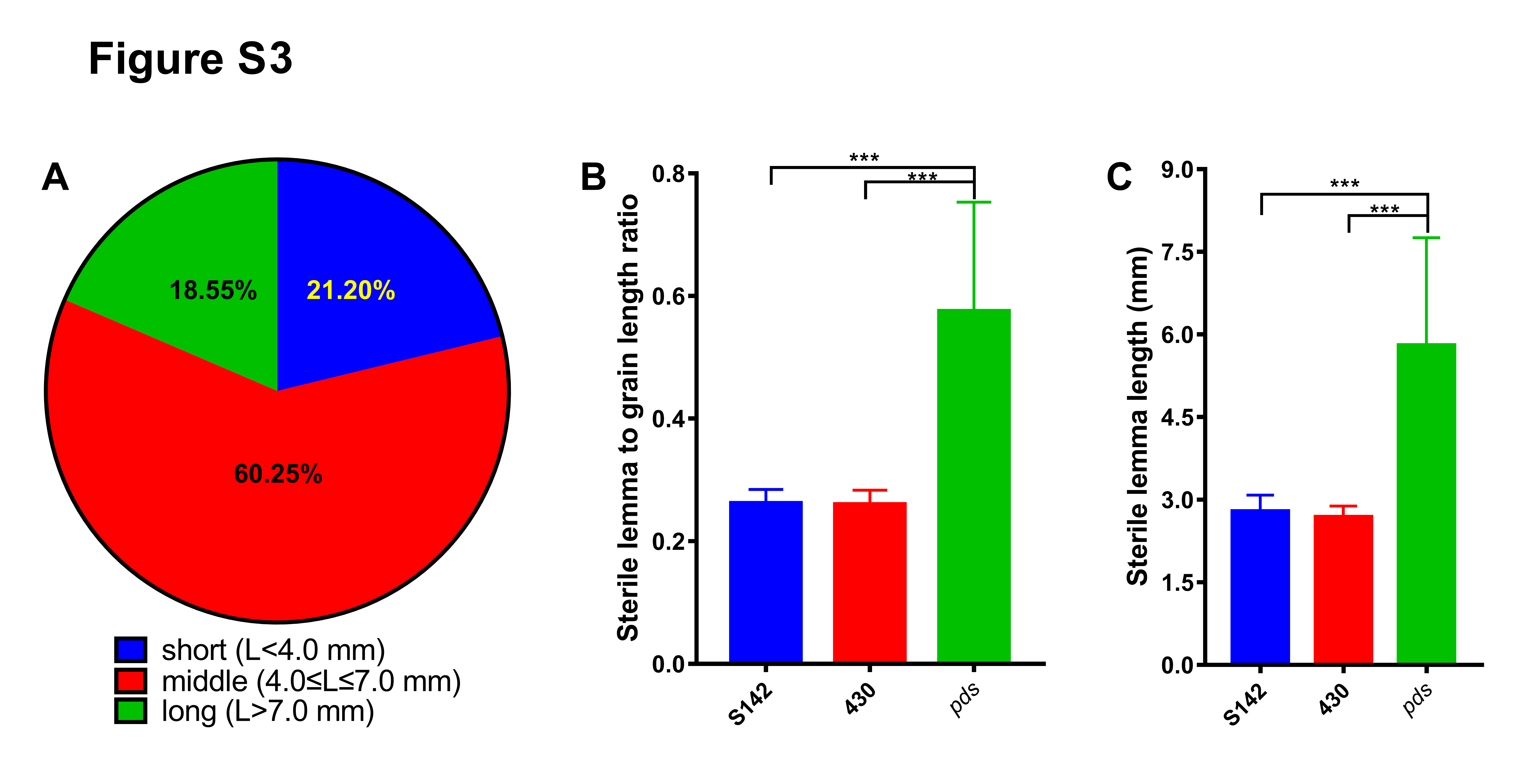

Supplement: Supplementary file 3 — Figure S3. Phenotypes of the elongated sterile lemmas in the spikelets of the pds line. (A) Percentages of three types of elongated sterile lemma. (B) Comparison of the sterile lemma to grain length ratio of S142, 430, and pds, showing the significant elongation of sterile lemmas in pds. (C) Comparison of sterile lemma lengths in S142, 430, and pds, showing the significant elongation of sterile lemmas in pds. Error bars in (B and C) indicate the mean ± sd; ∗∗∗Significant difference at P < 0.01 compared with the controls according to Student’s t-test in (B and C). (TIF 3150 kb) [file 12870_2019_1805_MOESM3_ESM.tif]

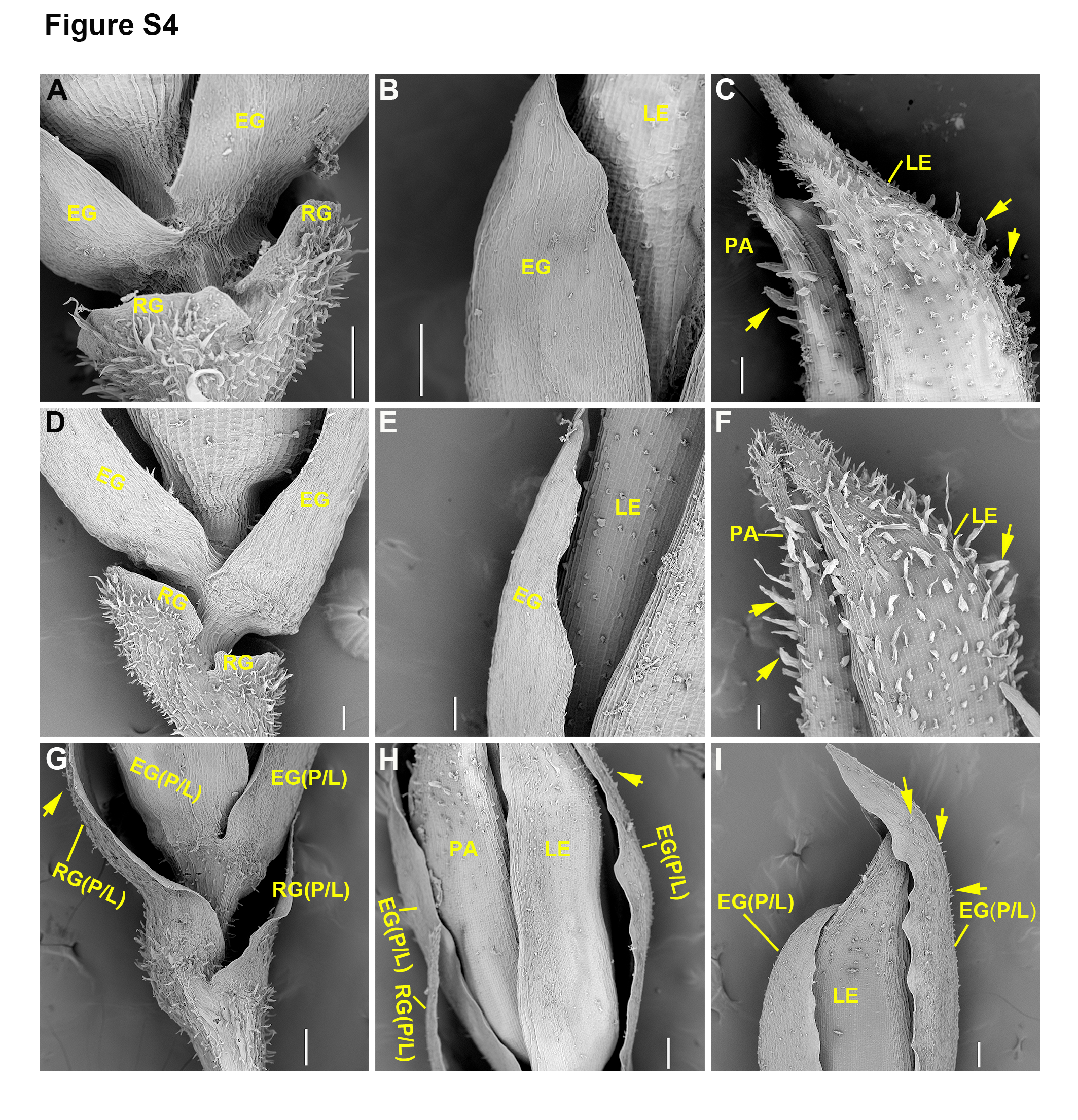

Supplement: Supplementary file 4 — Figure S4. Scanning electron microscopy (SEM) analysis of the development of the empty glume and rudimentary glume in the spikelets of S142, 430, and pds plants. (A) Empty glume and rudimentary glume of S142. (B) Epidermal surface of the empty glume of S142. (C) Epidermal surface of the palea and lemma of S142. (D) Empty glume and rudimentary glume of 430. (E) Epidermal surface of the empty glume of 430. (F) Epidermal surface of the palea and lemma of 430. (G) Empty glume and rudimentary glume of pds. (H) Epidermal surface of the empty glume of pds. (I) Epidermal surface of the palea/lemma-like structures of pds. PA, palea; LE, lemma; EG, empty glume; RG, rudimentary glume; ST, stamen; STI, stigma; P/LL, palea/lemma-like structure. The yellow arrows in (E, F, G, I) designate the trichomes on the surfaces of the spikelet organs. Bars = 100 μm in all panels. (TIF 16971 kb) [file 12870_2019_1805_MOESM4_ESM.tif]

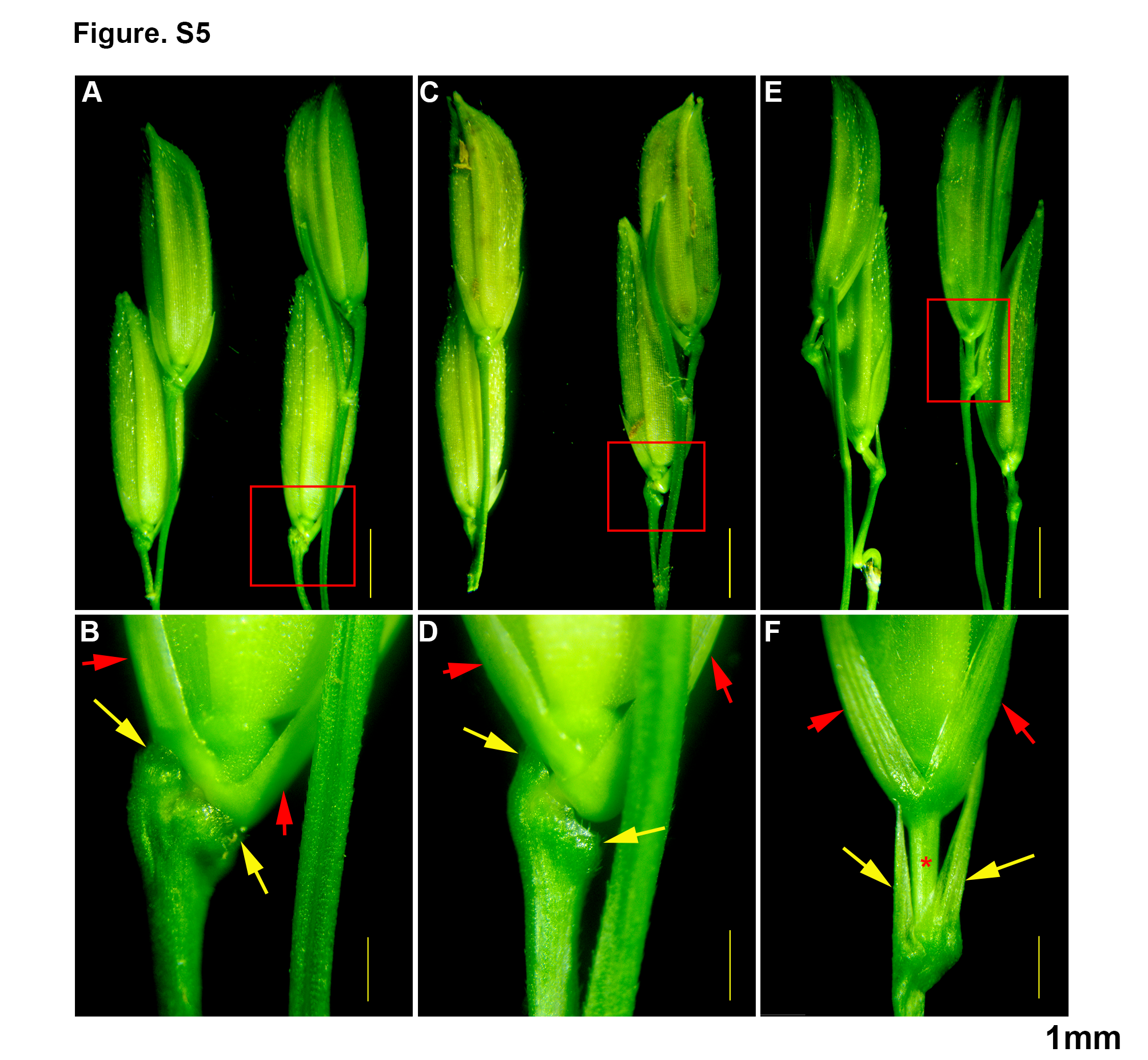

Supplement: Supplementary file 5 — Figure S5. Morphological phenotypes of the spikelets of S142, 430, and pds plants, showing the alteration of the rudimentary glumes in the pds line. (A, C, E) Secondary rachis branches of S142, 430, and pds plants with mature spikelets, respectively. (B, D, F) Magnifications of the boxed regions in (A, C, E), respectively. The yellow arrows in (B, D, F) show the rudimentary glumes of the spikelets. The red asterisk in (F) shows an elongated pedicel. Bars =1 mm in (A-F). (TIF 5364 kb) [file 12870_2019_1805_MOESM5_ESM.tif]

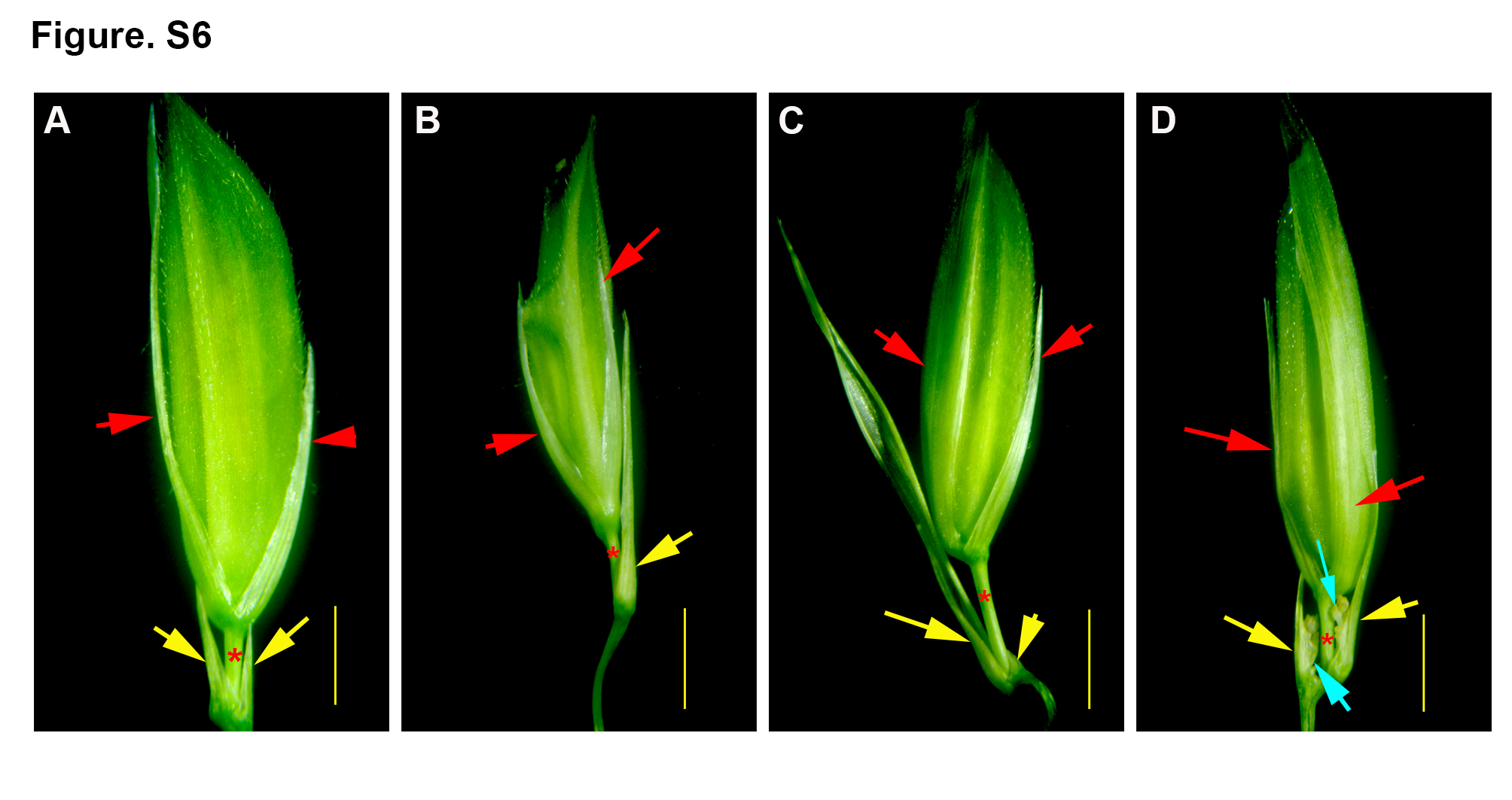

Supplement: Supplementary file 6 — Figure S6. Morphology of pds spikelets, showing the different types of elongation of the sterile lemmas, rudimentary glumes, and pedicels. (A) Spikelet with two nearly equal rudimentary glumes and two different lengths of sterile lemmas. (B) Spikelet with two nearly equal sterile lemmas and one elongated rudimentary glume. (C) Spikelet with two different lengths of rudimentary glumes and two different sterile lemmas. (D) Spikelet with two different lengths of rudimentary glumes, two different lengths of sterile lemmas and plantlets in the axils of the rudimentary glumes. The yellow and red arrows in (A-D) represent the rudimentary glumes and sterile lemmas, respectively. The light blue arrows in (D) show the plantlets in the axils of the rudimentary glumes. Bars = 1 mm in (A-D). (TIF 2248 kb) [file 12870_2019_1805_MOESM6_ESM.tif]

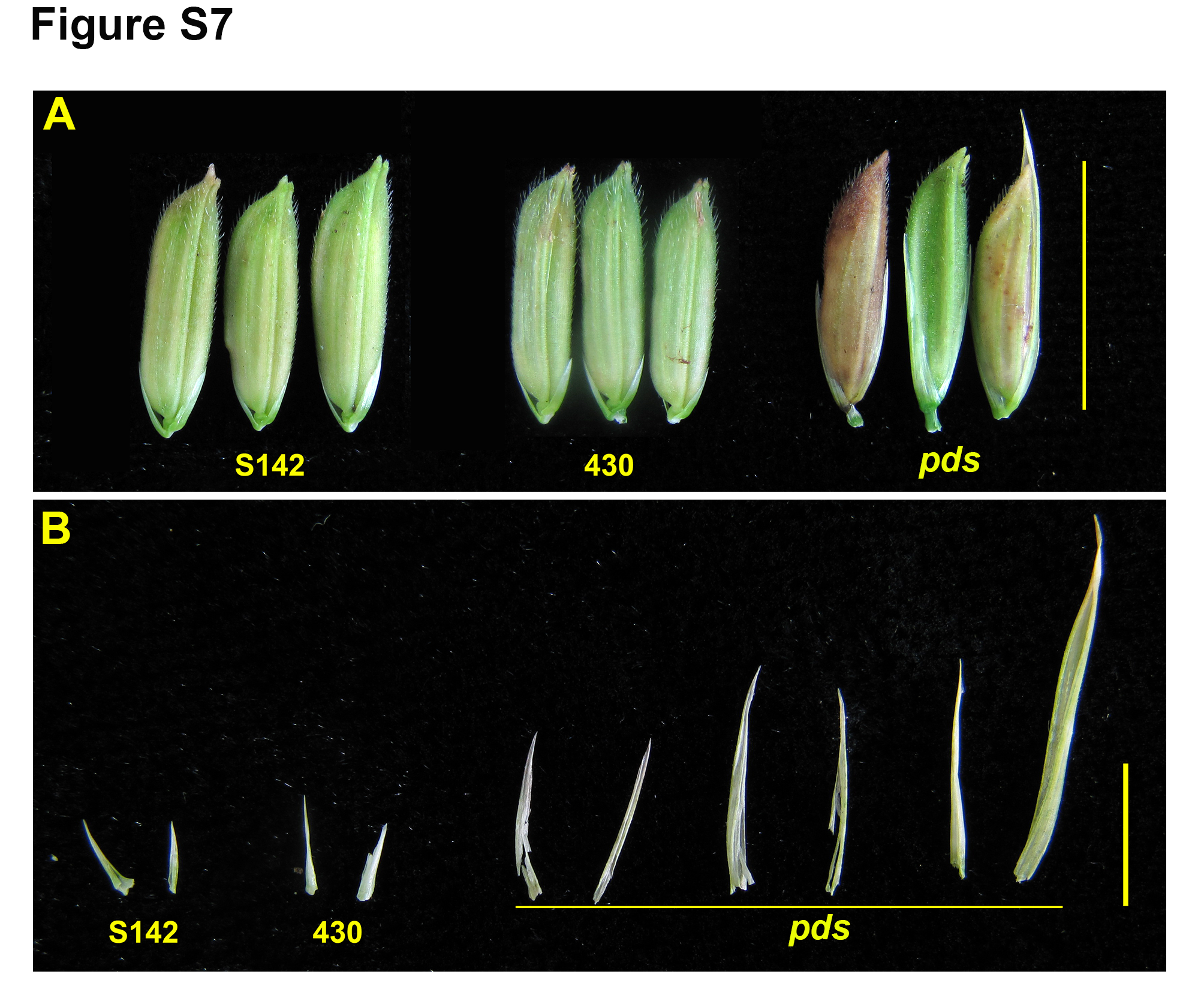

Supplement: Supplementary file 7 — Figure S7. Seed morphology of S142, 430, and pds plants. (A) Seeds of S142, 430, and pds. (B) Empty glumes of the seeds of S142, 430, and pds. Bars = 1 cm in (A), 3 mm in (B). (TIF 10152 kb) [file 12870_2019_1805_MOESM7_ESM.tif]

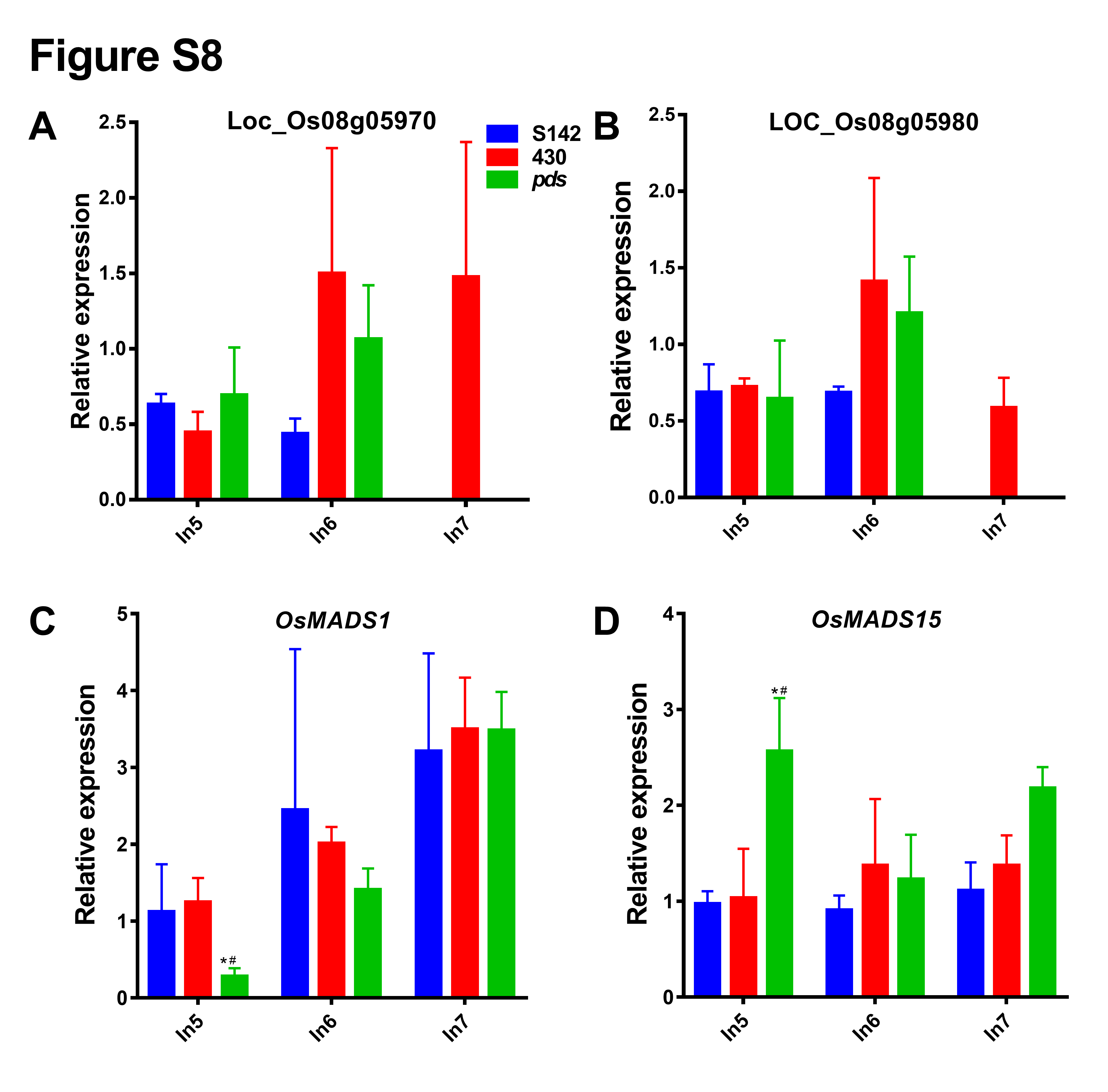

Supplement: Supplementary file 8 — Figure S8. The expression pattern analysis of pds1 candidate genes and spikelet development related genes at spikelet development stage In5, 6, and 7 of S142, 430, and pds plant. (A) The expression level of candidate gene Loc_Os08g05970. (B) The expression level of candidate gene Loc_Os08g05980. (C) The expression level of spikelet development related gene OsMADS1. (D) The expression level of spikelet development related gene OsMADS15. (TIF 1983 kb) [file 12870_2019_1805_MOESM8_ESM.tif]
