# Supplementary material for: The rice pds1 locus genetically interacts with partner to cause panicle exsertion defects and ectopic tillers in spikelets
Source: BMC Plant Biol. 2019 May 15;19:200. doi: 10.1186/s12870-019-1805-z (PMC6521401; doi:10.1186/s12870-019-1805-z)
Supplement: Supplementary file 9 — Table S1. The primer sequences of related genes using for qPCR. Table S2. The descriptive statistical analysis of the length of sterile lemma and rudimentary glume. Table S3. Grain yield related traits of S142, 430, and pds rice plants. (DOCX 23 kb) [file 12870_2019_1805_MOESM9_ESM.docx]

Table S1 The primer sequences of related genes using for qPCR

| **Primer name** | **RGAP_ID** | **Forward Primers (5'-3') Reverse Primers (5'-3')** | |
| --- | --- | --- | --- |
| ***q05940*** | LOC_Os08g05940 | CATACTCACCTACCTCACCATC | AGATGTTGGGCTATAATCTGGG |
| ***q05950*** | LOC_Os08g05950 | CACGCCACCAGCCGCAACCA | TTCCTCCGTCGTCCTCGCCTCC |
| ***q05960*** | LOC_Os08g05960 | TGTGCGATCAAGATCAGATCAT | CCCTCTTGCTCTCCTTGTATC |
| ***q05970*** | LOC_Os08g05970 | AGCAGGAAGAAGATGAACAAGA | TATCATACACACTAGCTGCTCG |
| ***q05980*** | LOC_Os08g05980 | CGACGACGATGAAGAGTAACTA | CCTATAAGTGGGTTCGGTTCAT |
| ***q05990*** | LOC_Os08g05990 | TATGCCGTCGCTGTTGGG | GCAAGCCAGTCGGTGATGTTC |
| ***OsMADS15*** | LOC_Os07g01820 | CTGAAGCGGATAGAGAACAAGA | CGGATTCAGCTGAAATAAGAGC |
| ***OsMADS1*** | LOC_Os03g11614 | TATTTTGCTAAGTACGTGCGTG | ACTTGTTACCACATCCAAAACG |
| ***G1*** | LOC_Os07g04670 | CGTCTACTTGCCATTTCTGTAC | GAGAAGCACAGACAAATACACG |
| ***Actin*** | LOC_Os11g06390 | GAGTATGATGAGTCGGGTCCAG | ACACCAACAATCCCAAACAGAG |

Table S2 The descriptive statistical analysis of the length of sterile lemma and rudimentary glume

|  | **Sterile lemma length** | | |  | **Rudimentary glume length** |
| --- | --- | --- | --- | --- | --- |
|  | **S142** | **430** | ***pds*** |  | ***pds*** |
| **Mean** | 2.79±0.29 | 2.69±0.19 | 5.80±1.95 |  | 5.07±4.39 |
| **Range** | 2.46-3.35 | 2.42-3.06 | 2.60-11.23 |  | 0.69-14.87 |
| **Kurtosis** | 0.03 | -0.34 | -0.18 |  | 0.13 |
| **Skewness** | 0.61 | 0.61 | 0.86 |  | 1.08 |

Notes: All data are given as mean±sd; S142 and 430 n=10, *pds* n=100; unit: mm.

Table S3 Grain yield related traits of S142, 430, and *pds* rice plants

| **Trait** | **S142** | **430** | ***pds*** | **S142/*pds* *p*-value** | **430/ *pds* *p*-value** |
| --- | --- | --- | --- | --- | --- |
| **PL** | 27.2±3.06 | 23.55±1.67 | 19.3±3.55 | <0.01 | <0.01 |
| **NPB** | 9.50±1.58 | 16.50±1.36 | 9.00±2.44 | >0.05 | <0.001 |
| **NSB** | 34.00±10.25 | 43.50±11.55 | 23.00±5.41 | <0.0001 | <0.0001 |
| **SSR (%)** | 86.67±8.03 | 69.97±11.56 | 16.17±13.77 | <0.0001 | <0.0001 |
| **NGP** | 195.05±29.00 | 193.13±58.83 | 8.03±4.95 | <0.0001 | 0.0001 |
| **GYP** | 54.49±12.09 | 27.67±12.45 | 2.06±1.17 | <0.0001 | 0.0016 |
| **GL** | 10.62±0.53 | 10.23±0.65 | 9.56±0.90 | ns | ns |

Notes: SSR, seed setting rate; GYP, grain yield per plant; NPB, number of primary branches; NSB, number of secondary branches; NGP, number of grain per plant; GL, grain length; PL, panicle length. All data are given as mean±sd, a student’s *t*-test was performed to generate *p*-values; S142 and 430 n=20, *pds* n=60.
